# Fault diagnosis of rolling bearing failures using a multi-stage e-CNN-GRU-SAM network

**DOI:** 10.1038/s41598-025-17008-y

**Published:** 2025-09-26

**Authors:** Santosh Bisoyi, Amit Kumar Rathi, Swarup Mahato

**Affiliations:** 1 Department of Civil and Infrastructure Engineering, Indian Institute of Technology Jodhpur, Jodhpur, 342030 Rajasthan India; 2 Rishabh Centre for Research and Innovation in Clean Energy, Indian Institute of Technology Jodhpur, Jodhpur, 342030 Rajasthan India; 3 Department of Mechanical and Production Engineering, Aarhus University, Aarhus, 8200 Denmark

**Keywords:** Fault diagnosis, Prognostic analysis, Type of fault, Rolling bearings, Convolution neural network, Gated recurrent unit, Segment anything model, Civil engineering, Energy infrastructure

## Abstract

This study presents a forensic diagnostic framework aimed at enhancing the early detection, fault classification and remaining useful life (RUL) prediction of rolling bearing failures. The proposed network integrates a novel three-stage machine learning formulation – (1) identification of health state using voting ensemble, (2) prognostic analysis via a hybrid convolutional neural network and gated recurrent unit (CNN-GRU), and (3) fault type identification through the segment anything model (SAM) based on time-frequency representations. The ensemble and CNN-GRU models are trained on both time- and frequency-domain features from vibration signals, while SAM leverages this data in visual sense through iterative masking for zero-shot spatial-temporal fault segmentation. Pre-processing techniques, including piecewise aggregate approximation and singular spectrum analysis, are used to denoise and compress the vibration response without impacting key statistical traits. The proposed e-CNN-GRU-SAM network demonstrates better accuracy in diagnosing fault types, predicting RUL and identifying root causes under different operational conditions. This is established using diverse operating benchmark datasets that simulate induced and real-world degradation scenarios for generalization. Thus, the proposed framework offers a comprehensive forensic analysis toolkit for diagnosis and prognosis of bearings.

## Introduction

Gearbox bearings are critical due to continuous deterioration, as they are susceptible to wearing of rolling metal parts, plastic deformation, fatigue failures, and inner and outer raceway faults^1,2^. The uncertainty in the forces often results in abnormal shaft vibrations which amplifies the deterioration in the gearbox bearings^3^. This limits the service life of bearings and their sudden failures result in unexpected downtime of operations which impacts production, energy harvesting, scheduled maintenance, safety and reliability^4^. Thus, frequent inspections and maintenance scheduling of the gearboxes are required to ensure smooth operations, which escalates logistic expenses, especially for wind turbines in offshore locations^5^. As a solution, a robust bearing design or effective condition assessment is required. The present study focuses on improving the bearing fault forensic mechanism to predict health, remaining service life and type of fault.

In condition assessment, vibration based techniques are widely used, such as empirical mode decomposition (EMD)^6^, Hilbert-Huang transformation (HHT)^7^, wavelet transformation^8,9^ and their modified versions^10–12^. These techniques decompose the vibration signal to diagnose system degradation due to damage or fault. Recently, these techniques have been combined with machine learning (ML) models to determine classification of system failures^6–8,10,12^. Kankar et al. (2011)^8^ suggested the wavelet transformation with multiple ML models to classify the health of the roller bearings. It adopted the Morlet wavelet for the decomposition of the bearing vibration signal based on the minimal Shannon entropy criterion. Lei et al. (2011)^6^ proposed an ensemble EMD based wavelet transform and artificial neural network (ANN) method to predict fault in locomotive rolling bearings. The significant intrinsic mode function (IMF) using a kurtosis based method was adopted to identify the change in amplitude of the vibration response due to bearing fault. It considered both time and frequency domain features to detect bearing health. The fault diagnosis was further improved using the complete ensemble EMD and piecewise aggregate approximation (PAA) to compress the vibration signal^13^. However, it suffered low computational efficiency due to the long signal which limits its application for real-time fault diagnosis. Wang et al. (2022)^10^ assessed fault based on adaptive empirical wavelet transform (EWT) and convolutional neural network (CNN). The EWT has also been used with particle swarm optimization (PSO) and maximum correlation kurtosis deconvolution (MCKD) based on the maximum kurtosis value for the minimum entropy^14^. Moreover, a recurrent neural network (RNN) model based on long short-term memory (LSTM) was proposed for the detection of rolling element failures^15^. It was further improved by the bi-directional LSTM (bi-LSTM) approach in conjuncture with a discrete wavelet transform for early failure detection^16^. However, the major issue lies in the lack of an appropriate threshold value to identify bearing health for general application and early detection.

Similar approaches have been adopted in identifying fault type types of bearings (such as inner raceway fault, outer raceway fault, cage fault, rolling element fault etc.) based on different ML models. One of the methods to identify the defect type in rolling bearings is MCKD^17^ using time signal data. It has been further improved by combining the complete ensemble EMD^18^ to decompose the vibration signal for better performance. Rezamand et al. (2020)^19^ proposed ordered weighted averaging operator to analyze the outer and inner raceway faults in bearings. The signals were denoised using the discrete wavelet transformation followed by the ordered weighted average to determine the significance of the time domain features. It was observed that the outer race bearing failure can be detected based on the variance of the time signal data, whereas the inner race failure can be identified using the root mean square (RMS) and peak-to-peak features of the signal. Another approach is to convert the vibration signals into a series of 2D images based on spectrogram^20^, scalogram^21–23^, Gramian angular field^24,25^ etc., which can be further trained for prediction. Wen et al. (2017)^20^ trained spectrogram images with deep learning (DL) based LeNet-5 to predict the type of bearing failure. It yields exhaustive computational cost in training due to different failures and limitations with image size. Other DL models such as AlexNet, GoogLeNet, ResNet50, EfficientNet-B0 and InceptionV3^26–28^ have been used to diagnose bearing defects and machine faults under constant rotational speed. However, these models often require significant computational cost due to deep learning networks^29^. Ullah et al. (2024)^22^ performed a general machine fault diagnosis using a modified AlexNet with three residual CNN blocks to extract spatial-temporal features. The sensitive features were filtered using ant colony optimization and support vector machine (SVM) to classify the machine faults. Siddique et al. (2024)^30^ employed CNN to extract temporal features of centrifugal pump using acoustic data. It detected the leakage in the pump using ANN based on t-distributed stochastic neighbor embedding (t-SNE) from CNN. Zaman et al. (2025)^31^ proposed transfer learning to identify the fault types in machine. Siddique et al. (2025)^23^ added multi-head attention in bi-LSTM to identify the bearing fault type. In case of variable speeds, the convolutional block attention module^32^ and graph neural networks^33^ have been used to predict the fault type from nonstationary signals. Wang et al. (2025)^34^ proposed a phase entropy based approach using SVM for bearing fault diagnosis. It was further improved based on a few-shot learning with multi-scale perception and Gramian angular summation field was used to extract the phase entropy features^24,25^. Zhao et al. (2024)^32^ proposed CTNet based on the convolutional block attention module with residual encoder-decoder and linear chirplet transform for variable rotational speed data. Zhao et al. (2025)^33^ suggested the application of multiple graph neural networks to predict the type of fault with incomplete data. However, these methods require limiting cross-terms in the time-frequency features which may not be necessary for applications in constant rotational speed based faults and may attract more computational time. Hence, the present study focuses on developing methods that require less computational time without marginalizing the accuracy of the prediction. At times, CNN based models have performed well with improved accuracy without significant addition of the computational burden on the training process^21,35^. Also, sometimes the dimension of the problem is reduced in the training process, which may result in loss of information and the selection of the input size that affects accuracy^29^. Overall, a generalized and efficient formulation is required for the prediction of types of bearing failure.

In addition to the health classification and prediction of fault type, the failure diagnosis requires estimation of service life of the bearings. Formulating a precise physical model to predict the remaining useful life (RUL) is a cumbersome process and requires approximations^36^. To address this issue, Yu (2012)^37^ suggested a combination of dynamic principal component analysis (PCA) and hidden Markov model (HMM) to monitor bearing health. It highlighted the significance of features from time, frequency, envelope-based frequency and time-frequency domains (especially RMS, square mean root and wavelet energy). However, these features exhibited substantial inconsistency in performance when applied individually. Saidi et al. (2017)^38^ proposed a spectral kurtosis and support vector regression based RUL estimation framework using the time domain features such as kurtosis and peak-to-peak value in early detection of faults. They studied the effect of noise on the basis of the sensitivity of the mean and skewness of the time response. Li et al. (2018)^39^ proposed modifications in the data-driven prognostic method using deep CNN and a time window approach. The vibration signal was normalized to train the DL model with prior knowledge, but this yielded a higher value of root mean square error (RMSE). Similarly, deep adversarial neural network (DANN)^40^, residual neural network framework with multiscale attention mapping (ResNet-MA)^41^, self-attention temporal CNN^42^, self-attention augmented convolution GRU network (SACGNet)^43^, GRU-deep autoregressive model (GRU-DeepAR)^44^, and convolution attention mechanism and temporal convolution network (CAMTCN)^45^ were proposed to improve the prediction of rolling bearing RUL. The combination of the self-attention mechanism and the convolution framework assisted in the assignment of larger weights to essential features (related to the time and frequency domains) without neglecting local information using the Euclidean distance for similarity calculations. This results in better accuracy, but the integration of the self-attention mechanism with the neural network increases the computational cost in training. Also, the models trained on local information may yield overfitting and eventually, error in estimation using new data. Prasad et al. (2023)^46^ prescribed PAA with a sparse encoder based ML model, where the pre-processing in smaller time window size resulted in efficient statistical moment estimation without incurring significant errors. They also proposed a health indicator index based on the variance of the time signal including the service life cycle phases of rolling bearing (i.e., normal, incipient and severe) for better prediction. In addition to PAA, Gupta et al. (2023)^47^ suggested singular spectrum analysis (SSA) and autoencoder based ML model with gated recurrent unit (GRU) to identify the time position indicating the change in bearing health (i.e., change point). Chen et al. (2024)^48^ introduced a dilated inception network (DiIncepNet) with an integrated gated convolutional unit and temporal convolutional networks (TCN) to predict RUL. Li et al. (2024)^49^ proposed an image-based RUL prediction for rolling bearings. The vibration signals were converted into scalograms using the Morlet based continuous wavelet transform and the predictions were performed using causal dilated convolution based residual densely connected network with channel attention (CARDenseNet). However, it lacked the identification of the change points and can potentially incur an increased training time due to the dense learning network^50,51^. In early detection of the change point, Zhao and Pan (2024)^52^ introduced Gaussian derivatives with EMD and LSTM. However, the pre-processing of vibration data using EMD may result in mode mixing to restrict the accuracy of RUL estimation.

The aforementioned literature review suggests that the existing methods are computationally inefficient and there is scope for improvement in the prediction accuracy under forensic analysis. Additionally, these methods either predict the faults, estimate the RUL and/or identify the type of damage. Hence, a single unified framework to address all these critical aspects requires further exploration for bearing condition assessment. The objectives of the present study can be summarized as follows:

- Development of a single forensic framework to detect bearing health, failure type and prediction of the remaining service life of bearings.
- Improvement in deep learning network for an accurate bearing fault prognosis incorporating time and frequency analysis.
- Propose a generalized network for bearing fault diagnosis using visual data based learning without marginalizing critical information.

Based on these objectives, the present study proposes a novel framework to address the challenges associated with the diagnosis and prognosis of the bearing failure. This will help reduce the downtime of machines with rolling bearings (such as wind turbines, milling machines, centrifugal pumps etc.) by facilitating preventive maintenance^47^. The detailed framework for the condition assessment of bearings to classify health, predict RUL and identify the type of failure is discussed in the following sections.

## Input parameter extraction

Long vibration data often results in computational burden in analyzing and training the deep learning networks. To address this issue, efficient pre-processing and noise reduction techniques are required to distill the vibration signals without losing valuable information. This section illustrates the pre-processing techniques adopted in the study.

### Pre-processing of vibration signal

The raw vibrational data are recorded at a specific sampling frequency which may hide abrupt shifts and hence, it is imperative to employ robust pre-processing techniques to enhance the signal-to-noise ratio^36^. In this study, two such techniques, *viz.* PAA and SSA are adopted sequentially as discussed further.

#### Piecewise aggregate approximation technique

PAA streamlines time series data and preserves global as well as local characteristics^53^. It partitions the original time series $\varvec{Y}$ into smaller segments or sub-series of uniform length *w* (i.e., window size). The mean value of each segment is computed and the resultant time series is formed using the aggregation of these mean values to reflect the behaviour at a selected resolution. The granularity of this representation is governed by the selected scale to determine the length of the segment. Hence, the time series data $\varvec{Y} = [y_1, y_2, ..., y_n]$ is converted to a reduced time series $\varvec{X} = [\varvec{x}_1, \varvec{x}_2, ..., \varvec{x}_l]$, where *l* denotes the number of segments. Each reduced time series segment $\varvec{x}_j$ is the mean of a non-overlapping segment of $\varvec{Y}$ as given by^53^

1 $$\begin{aligned} \varvec{x}_j = \frac{l}{n} \sum _{i=\frac{n}{l}(j-1)+1}^{\frac{n}{l}j} y_i \hspace{5mm}\forall \hspace{2mm} j \in \{1,2,....,l\} \end{aligned}$$

In the next step, SSA is performed to reduce the effect of noise in the reduced time series data.

#### Singular spectrum analysis

SSA is a statistical technique used to estimate the spectral features of the signal. It is helpful when ambient noise may be present in the signal to reduce the clarity by mixing different signal components. The application often improves the signal-to-noise ratio and reduces the occurrence of false alarms, especially when identifying the change points in vibration signals^54^. The analysis has four discrete steps – embedding, decomposition, grouping and diagonal averaging. Initially, a time series is arranged to construct a covariance matrix called the trajectory matrix. It is further decomposed into multiple components such as gradual changes in the patterns, oscillating parts and noise using singular value decomposition (SVD) technique. The third step involves classifying comparable elements to form groups. At last, the time series is reconstructed by projecting the primary component (from the decomposition process) onto the eigenvectors.

The time series $\varvec{x}_j$, as per Eq. 1, is transformed into the trajectory matrix $\varvec{X}_w$ with dimensions $w~\times d$, where $d = l-w+1$. It is assumed that $w>d, \forall \hspace{2mm} \varvec{x}\in \mathbb {R}$ in the present application. The construction of $\varvec{X}_w$ results in a Hankel matrix which entails organization of the lagged vector such that all the anti-diagonal elements are identical, this process is called embedding. The matrix $\varvec{X}_w$ is decomposed using the SVD technique as

2 $$\begin{aligned} \varvec{X}_p = \varvec{U}_p\sqrt{\varvec{\lambda }_p}\varvec{V}^T_p \end{aligned}$$

where, the matrix $\varvec{X}_p$ is of rank one such that $[\varvec{X}_1, \varvec{X}_2, \ldots , \varvec{X}_d]$, $\varvec{\lambda }_p$ is the eigenvalues of $\varvec{X}_w \varvec{X}^T_w$, and $\varvec{U}_p$ denotes the eigenvector matrix of $\varvec{X}_w \varvec{X}^T_w$ which is often referred as the factor empirical orthogonal function. In Eq. 2, $\varvec{V}_p$ represents a square matrix of the principal components of $\varvec{X}_w$ with dimension *d*. The matrix $\varvec{X}_p$ can be split into several groups and the matrices are summed within each group. Let matrix $\varvec{X}_A := \varvec{X}_{a_1} + \varvec{X}_{a_2} + \ldots + \varvec{X}_{a_p}$ is associated with the set of indices $A = \{a_1, a_2, \ldots , a_p\}$. The split of the set indices $B = {1, 2, \ldots ,d}$ into the mutually exclusive subsets $A_1, A_2, \ldots ,$ and $A_m$ corresponds to

3 $$\begin{aligned} \varvec{X}= \varvec{X}_{A_1}+\varvec{X}_{A_2}+\ldots +\varvec{X}_{A_m} \end{aligned}$$

The process of selecting sets $A_1,A_2,\ldots ,$ and $A_m$ is referred to as the eigentriple grouping operation^54^. The contribution of the component $\varvec{X}_A$ to the expansion for a specific group *A* as per Eq. 3 is determined by the ratio of the eigenvalues given as $\sum \limits _{a\in A}\varvec{\lambda }_a / \sum \limits _{a=1}^d \varvec{\lambda }_a$.

Principal components are projected onto eigenvectors, i.e. diagonal averaging or Hankelization, to represent time series $\varvec{Z}$ in $\varvec{X}$. Finally, the original time series $\varvec{X}=[\varvec{x}_1, \varvec{x}_2, ..., \varvec{x}_l]$ can be decomposed into a summation of *m* series as

4 $$\begin{aligned} \varvec{x}_j=\sum _{q=1}^m \widetilde{\varvec{x}}_j \end{aligned}$$

where, $\widetilde{\varvec{x}}_j$ corresponds to $\varvec{z}_{ik}$. The $q^{th}$ term of the resulting series can be computed by taking the average of $\varvec{z}_{ik}$ (i.e. an element of a matrix $\varvec{Z}$) across all *i* and *k* indices satisfying the condition $i + k = q + 2$. The primary significance of SSA is the effectiveness of separability in recognizing and segregating various time series segments from each other. In case of real-time applications, a recursive variant of SSA is used to assist the direct reading of the recorded time series from the sensors and noise reduction.

**Fig. 1:**
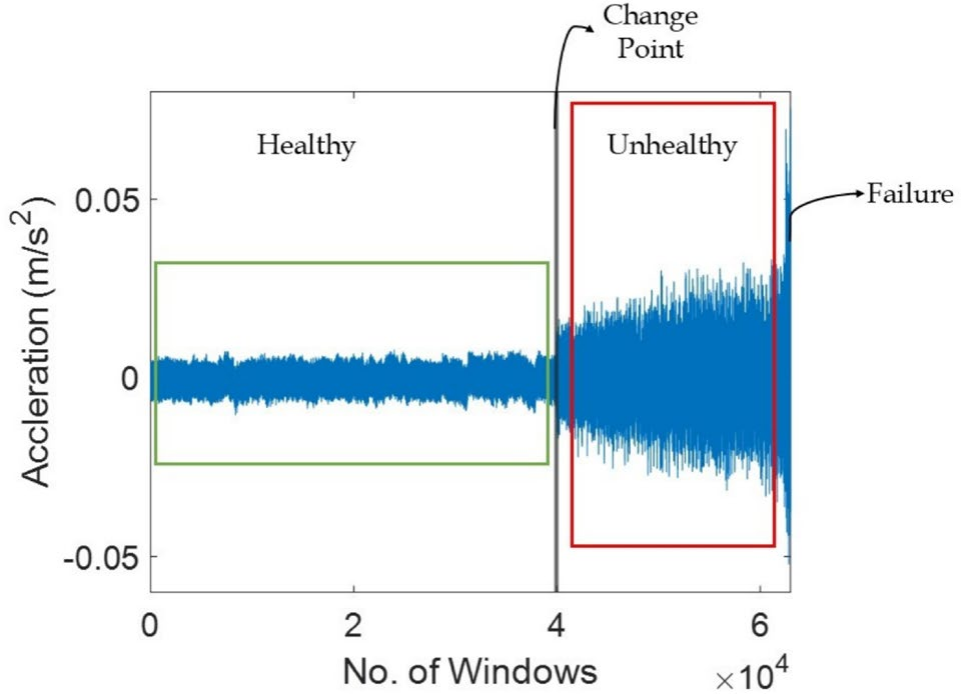
Vibration signal illustrating different phases of health in a bearing.

**Table 1:** Description of the time and frequency domain features of the vibration signals.

**Parameters**	**Formula**	**Description**
Mean $\mu$	$\frac{1}{w} \sum _{i=1}^w \varvec{x}_{j,i}$	Provides the average value of a dataset
Variance $\sigma ^2$	$\frac{1}{w} \sum _{i=1}^w (\varvec{x}_{j,i}-\mu _j)^2$	Measures the spread or variation of the data
Peak-to-peak (PP)	$\max (\varvec{x}_{j})-\min (\varvec{x}_{j})$	It is the range of the data using the maximum and minimum values
Root mean square (RMS)	$\sqrt{\frac{1}{w} \sum _{i=1}^w \varvec{x}_{j,i}}$	Evaluates denseness of the dataset around the mean
Skewness *S*	$\frac{1}{w}\sum _{i=1}^{w}(\frac{\varvec{x}_{j,i}-\mu _j}{\sigma _j})^3$	Measures spread of the dataset about the mean
Kurtosis *K*	$\frac{1}{w}\sum _{i=1}^{w}(\frac{\varvec{x}_{j,i}-\mu _j}{\sigma _j})^4$	Describes the shape of a probability distribution and measures the degree of peak distribution
Fifth moment $\gamma _5$	$\frac{1}{w}\sum _{i=1}^{w}(\frac{\varvec{x}_{j,i}-\mu _j}{\sigma _j})^5$	Evaluates the hyper-skewness
Sixth moment $\gamma _6$	$\frac{1}{w}\sum _{i=1}^{w}(\frac{\varvec{x}_{j,i}-\mu _j}{\sigma })^6$	Estimates the hyper-tailedness
Seventh moment $\gamma _7$	$\frac{1}{w}\sum _{i=1}^{w}(\frac{\varvec{x}_{j,i}-\mu _j}{\sigma _j})^7$	Measures the shape and asymmetry of a probability distribution
Shape factor (SF)	$\frac{RMS _j}{\mu _j}$	Describes the shape of a pulse or periodic signal
Crest factor (CF)	$\frac{\max (\varvec{x}_{j})}{RMS _j}$	Estimates the peak-to-average power ratio of a signal
Impulse factor (IF)	$\frac{\max (\varvec{x}_{j})}{\mu _j}$	Identifies the presence and impact of extreme values in the data
Latitude factor (LF)	$\frac{\sigma _j}{\mu _j}\times 100$	Calculates the degree of variation
Spectral mean $\mu _{\omega }$	$\frac{1}{w} \sum _{i=1}^w F_{j,i}$	It is the average value of a dataset in frequency domain
Spectral variance $\sigma _{\omega }^2$	$\frac{1}{w} \sum _{i=1}^w (F_{j,i} - \mu _{\omega _j})^2$	Measures the spread of the data in frequency domain
Spectral skewness $S_{\omega }$	$\frac{1}{w} \sum _{i=1}^{w}(\frac{F_{j,i} - \mu _{\omega _j}}{\sigma _{\omega _j}})^3$	Estimates spread of the dataset in frequency domain about the mean
Spectral kurtosis $K_{\omega }$	$\frac{1}{w} \sum _{i=1}^{w}(\frac{F_{j,i} - \mu _{\omega _j}}{\sigma _{\omega _j}})^4$	Describes the shape of a probability distribution in frequency domain and measures the degree of peak distribution

### Time and frequency parameters

The aforementioned pre-processing divides the time series into multiple small datasets $\varvec{x}_j$. The key input parameters can be estimated to train the ML and/or DL models for fault identification and prediction of remaining life. Statistical analysis of a dataset estimates the moments (i.e., mean, variance, skewness, kurtosis etc.) to indicate its critical parameters which may be overlooked otherwise^55^. In addition, it estimates the shape and scale of the data distribution. These moments are particularly sensitive to anomalies in the vibration signal which, in turn, helps in its detection. The emergence of failure in a system such as bearing, often indicates change in the characteristics of the vibration signal^55^. Hence, the estimation of moments as

5 $$\begin{aligned} \gamma ^s_{o,j} = \frac{s}{w} \sum _{i=1}^{w} \left[ \left( \frac{{\varvec{x}}_{j,i}-{\mu }_j}{\sigma _j} \right) ^s \right] ^o \hspace{5mm} \forall \hspace{2mm} {s = 1,2,....} \end{aligned}$$

helps to identify these irregularities in the time series data for early diagnosis of bearing defects. In the above equation, $\varvec{x}_{j,i}$ represents the $i^th$ element of the $j^th$ time series segment, *o* denotes the order of the moment and *s* is the scale of the time series. Based on the literature review^19^, in this study a total of 13 time domain features and four frequency domain characteristics are estimated for every time series window. The description of these parameters and formulations is presented in Table 1. The size of the dataset for each time series window is *w* and the Fourier transform function *F* is calculated as^9,11^

6 $$\begin{aligned} F_j(\omega )=\int _{-\infty }^{\infty } \varvec{x}_j(t)e^{-\iota \omega t} \,dt \end{aligned}$$

where, *t* and $\omega$ denote time and frequency, respectively.

**Algorithm 1:**
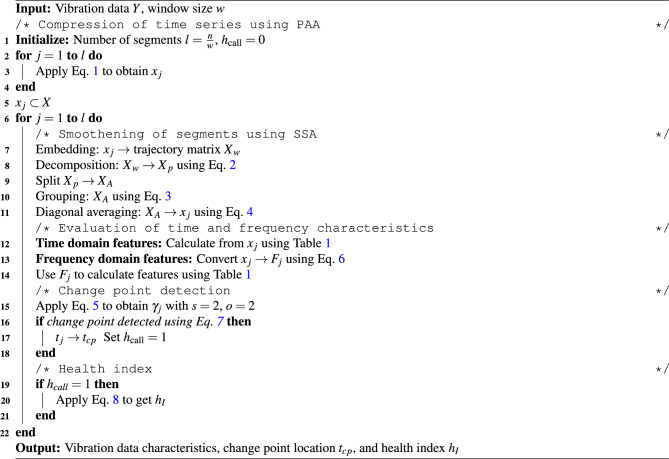
Pre-processing and statistical evaluation.

Additionally, the change point of the time series is also identified which marks the initiation of system deterioration as shown in Fig. 1. The second central moment (i.e., $s = 2$) is chosen to detect the change point of the time series as an optimal scale value^46^. The change point detection often suffers from the identification of false alarms^47^ and eventually requires a threshold value to avoid such instances. This threshold value $c_{th}$ can be evaluated using the slope of the moments of consecutive time series segments, i.e. $\gamma _{j-1}$ and $\gamma _j$. Hence, the change point identification condition is expressed as

7 $$\begin{aligned} \left( \frac{t_{j-1}-t_1}{ \gamma _{j-1} - \gamma _1} \right) \left( \frac{ \gamma _{j} - \gamma _{j-1}}{t_{j}-t_{j-1}} \right) \ge c_{th} \end{aligned}$$

where, the threshold value $c_{th} \in [1, 6]$. The first instance of a time step that satisfies Eq. 7 is the change point $t_{cp}$ of the time series. Since the change point, the condition of the system starts to deteriorate significantly. The health of the bearing is assumed to be fit until this time followed by a gradual decline in its performance. An index $h_I$ is defined to estimate the progressive decline in the health of the system. In this study, a linear decline in bearing health is adopted as^47^

8 $$\begin{aligned} h_I(q) = 1 - \frac{q - t_{cp}}{t_f - t_{cp}} \end{aligned}$$

where, $h_I \in [0, 1]$ with the unity value indicates that there is no fault, and the failure point of the dataset $t_f$ has $h_I = 0$, i.e. complete failure. The pre-processing of the raw vibration signal and the evaluation of the aforementioned features are sequentially explained in Algorithm 1.

## Formulation of Learning Networks

As mentioned earlier, this study focuses on the forensic prediction of fault, its type and estimation of the service life after damage in the bearings. Hence, different learning networks are required to effectively establish these distinct input-output relations. Three different models such as voting ensemble^56^, GRU with CNN^44,57^ and a recently developed segment anything model (SAM)^58^ with deep networks are considered in the present study as illustrated in this section.

**Fig. 2:**
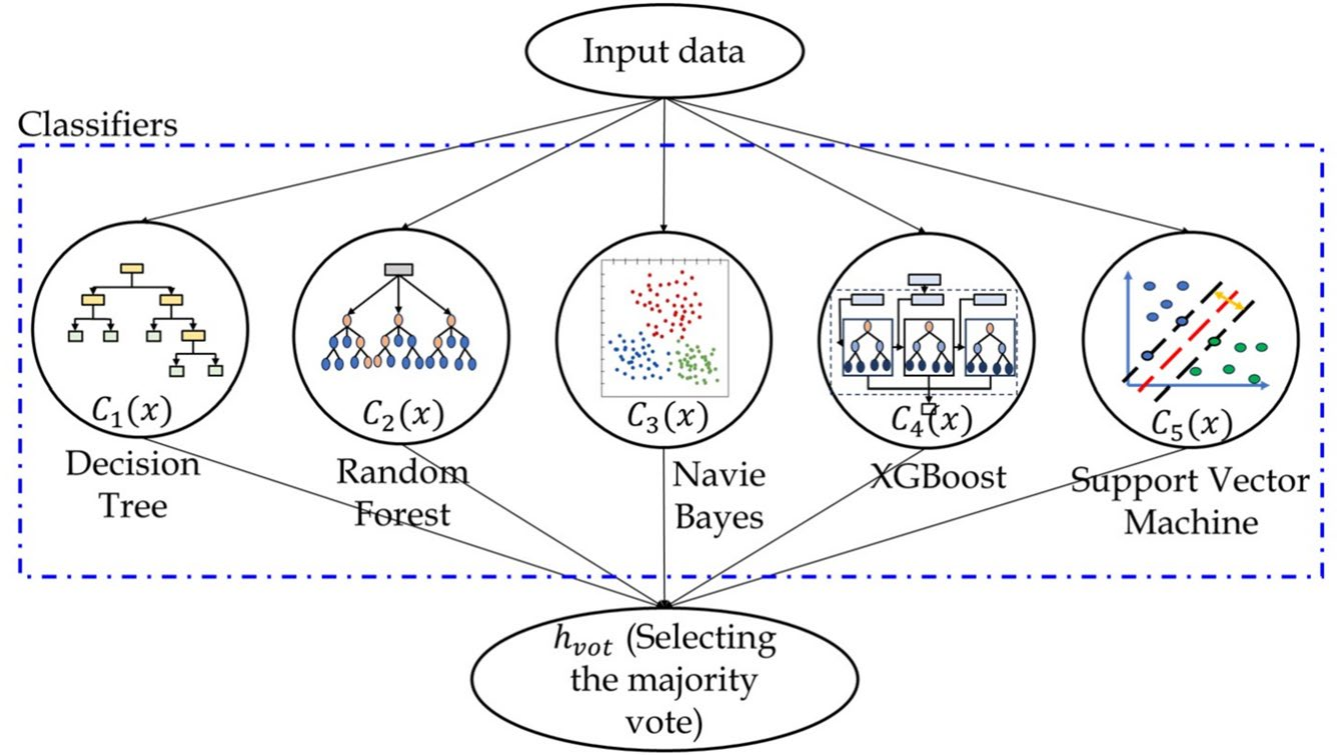
Typical architecture of voting ensemble based learning model with different classifiers.

**Algorithm 2:**
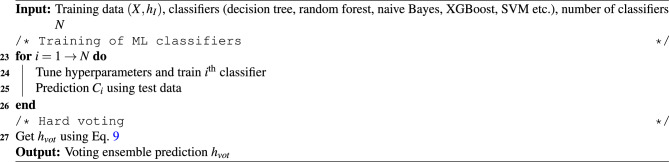
Voting ensemble learning framework.

### Voting ensemble learning model

The voting ensemble integrates the predictions of multiple distinct learning models to produce a final prediction^56^. Each of these separate models, termed as *classifier*, casts a *vote* for a particular outcome within this ensemble of ML models. The outcome (i.e., *class*) with the majority of votes is selected as the final prediction of the ensemble learning. It can include different learning algorithms with combinations of distinct set of parameters. This approach leverages diverse perspectives from various models or classifiers which enhances its overall performance and applicability. Fig. 2 shows a schematic operation of the voting ensemble based learning model considering different classifiers. The majority voting, also known as the *hard voting* $h_{vot}$ of the ensemble learning can be expressed as^56^

9 $$\begin{aligned} h_{vot} = mode \{C_1(x),C_2(x),\dots ,C_N(x)\} \end{aligned}$$

where, $C_i$ denote the ML classifiers such as decision tree, random forest, naive Bayes, extreme gradient boosting (XGBoost), SVM and so on^56^. In Eq. 9, $mode \{\bullet \}$ represents the counting of similar classes from the different classifiers $C_i$. The class with the highest votes is selected as the outcome of the ensemble learning model. The votes are evaluated based on the performance metrics such as precision, recall and F1 score. In this study, these measures are subjected to binary classification (i.e., positive and negative classes) which follow four types of outcomes – true positive (TP), true negative (TN), false positive (FP) and false negative (FN). If the model accurately predicts the positive class from the training dataset, then all such cases are added to get the number of true positive events. This indicates the proficiency of the classifier to identify the positive class. The true negative is defined as the ability of the classifier to accurately exclude instances belonging to the negative class. The false positive is when a model erroneously predicts the positive class which indicates its ability to predict false alarms and hence, such cases must be minimized. Lastly, the outcome is called false negative when the classifier incorrectly predicts the negative class which reflects poor learning. Table 2 presents the performance metric and here, $n_i$ indicates the number of *i* outcomes. A step-wise process of the voting ensemble based learning is presented in Algorithm 2.

**Table 2:** Evaluation indices for binary classification based learning models^56^.

**Criteria**	**Formula**	**Description**
Precision	$\frac{n_{TP }}{n_{TP } + n_{FP }}$	This metric indicates the proportion of correct prediction of positive class from the total predicted positive classes.
Recall	$\frac{n_{TP }}{n_{TP } + n_{FN }}$	It is the proportion of correct prediction of positive class from the actual positive classes and also, referred as sensitivity.
F1 score	$2\times \frac{Recall \times Precision }{Recall + Precision }$	It is a weighted average of accuracy and recall metrics.

### Gated recurrent unit based convolution neural network

CNN is DL model that employs neural networks to primarily extract features from structured dataset such as the bearing vibration response. It helps in recognizing the dataset patterns where the neural network acts as a feed-forward flow and the weight-sharing structure makes the learning process similar to our biological neural network^57^. This reduces the no. of weights required to train the model and hence, decreases computational burden. In general, the CNN is composed of three major components – convolutional layer, pooling layer and fully connected layer^57^. Typically in CNN, the convolutional and pooling layers are present in tandem where the neurons of the convolutional layer exhibit selective connectivity with specific neurons of the other layer(s). It extracts features from the subsets of input data using convolution operations based on kernel functions^57^. This process involves mapping the convolution kernel window across the input dataset. The dimension of the filter, also known as kernel size, is used to extract features from the input data and it is represented as a matrix of size $H \times D$. In general, the convolution operations are linear and hence, require an additional activation function to cater nonlinearity between two consecutive layers. Often, activation functions are defined using sigmoid, hyperbolic and ramp [i.e., rectified linear Unit (ReLU)] functions^56^. The convolution network can be expressed using the activation function $\mathscr {F}$ as^57^

10 $$\begin{aligned} \mathscr {X}_j^{(n+1)} = \mathscr {F}(W_{e_j}^{(n+1)} \circledast \mathscr {X}^n + b_j^{(n+1)}) \end{aligned}$$

where, $\mathscr {X}_j^{(n+1)}$ denotes the $(n+1)^{th }$ characteristic of the output value in the $j^{th }$ layer. In the above equation, the matrix $W_{e_j}^{(n+1)}$ defines the weights of the $j^{th }$ convolution layer, $b_j^{(n+1)}$ is the bias term and $\circledast$ indicates the convolution operation.

The pooling layer filters the input features following the extraction process in the convolutional layer, as mentioned above. This layer serves the purpose of spatial merging (i.e., downsampling) to reduce the dimensionality of the feature map without impacting the characteristics of the dataset. In this study, the widely used pooling operations, such as average and maximum functions, are adopted. The average pooling layer is expressed as

11 $$\begin{aligned} \mathscr {Y}_j^{(n+1)} = avg ({\textbf {x}}_j^i(k)), \hspace{5mm} k \in \mathscr {C}_i \end{aligned}$$

and the maximum pooling (i.e. maxpooling) layer is evaluated using

12 $$\begin{aligned} \mathscr {Y}_j^{(n+1)}=\max ({\textbf {x}}_j^i(k)), \hspace{5mm} k \in \mathscr {C}_i. \end{aligned}$$

In Eq. 11 and 12, $\mathscr {Y}_j^{(n+1)}$ represents the element in the $j^{th }$ feature map of the $(n+1)^{th}$ layer after pooling, $\mathscr {C}_i$ denotes the $i^{th }$ pooling area and $x_j^i(k)$ is the element of the $i^{th }$ pooling area in the $j^{th }$ feature map of the $n^{th }$ layer. Following the above expressions, connections are established between the different convolutional and pooling layers. Every neuron establishes connections with all the neurons in the preceding layer. Hence, a fully connected layer has the capability to incorporate the detailed information obtained from either the convolutional or the pooling layer.

**Fig. 3:**
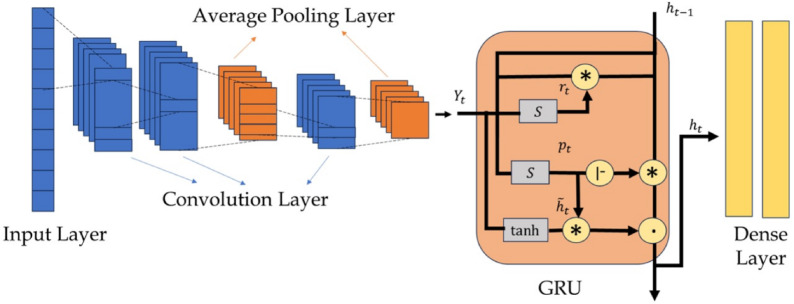
Architecture of the different layers in the combined CNN-GRU network.

**Algorithm 3:**
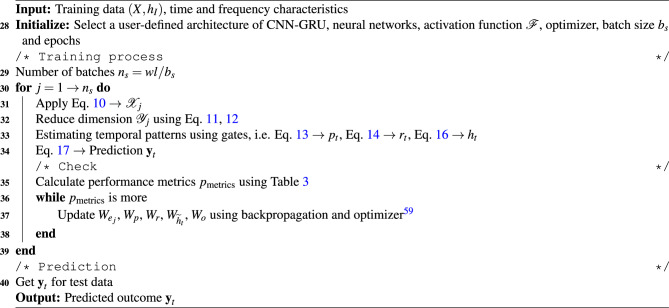
CNN-GRU learning framework.

GRU network is a type of RNN characterized by cyclic inter-neuronal connections^44^. It acquires and retains information based on selective learning using both long-term and short-term memories. This leads to a robust capability for feature extraction and therefore makes the technique attractive for a large dataset. The number of neurons in the input and output layers is governed by the respective dimension of the input and output spaces. The hidden layer(s) housing the memory cells encompass the primary functionalities of the GRU network. The learning of these cells depends on two logical cellular gates, *viz.* update $p_t$ and reset $r_t$. The update gate $p_t$ filters the information from the previous data (say time series) and accordingly, uses the relevant information in the present data. It is expressed as^44^

13 $$\begin{aligned} p_t = \mathscr {S}(W_p * [h_{t-1},\mathscr {Y}_t]) \end{aligned}$$

where, $h_{t-1}$ and $\mathscr {Y}_t$ represent the information of the previous data and the input of the current data, respectively. In the above equation, $\mathscr {S}(\bullet )$ denotes a sigmoid function [i.e., $e^{\bullet } / (1 + e^{\bullet })$]. Here, more information is selected for learning as the value of the update gate increases. The reset gate $r_t$ calculated by

14 $$\begin{aligned} r_t = \mathscr {S}(W_r * [h_{t-1},\mathscr {Y}_t]) \end{aligned}$$

is employed to regulate the extent of information from the previous data^44^. It is used by the candidate set $\widetilde{h}_t$ as

15 $$\begin{aligned} \widetilde{h}_t = \tanh (W_{\widetilde{h}_t} * [r_t . h_{t-1}, \mathscr {Y}_t]) \end{aligned}$$

to get the output information $h_t$ of the current data series following

16 $$\begin{aligned} h_t = (1 - p_t) . h_{t-1} + p_t . \widetilde{h}_t \end{aligned}$$

where, the dense layer output is determined using^44^

17 $$\begin{aligned} {\textbf {y}}_t = \mathscr {S}(W_o * h_t). \end{aligned}$$

In the above equations, $*$ and . denote matrix and element-wise multiplications, respectively. Also, $W_r$, $W_p$, $W_{\widetilde{h}_t}$ and $W_o$ are weight matrices associated with the reset gate, update gate, candidate set and output, respectively. The performance of this DL model is evaluated using prediction evaluation indices as described in Table 3. The proposed CNN-GRU network is shown in Fig. 3 and the flow is explained in Algorithm 3.

**Table 3:** Different evaluation indices for performance of prediction models.

**Criteria**	**Formula**	**Description**
Mean absolute error (MAE)	$\frac{\sum _{i=1}^{n} |{\textbf {y}}_i - {\textbf {x}}_i|}{n}$	This index is the average of the absolute differences between the predicted and actual values.
Root mean square error (RMSE)	$\sqrt{\frac{\sum _{i=1}^{n} ({\textbf {x}}_i - {\textbf {y}}_i)^2}{n}}$	It is the square root of mean square error and provides a measure of average prediction error in the original units.
R-square (R2) score	$1 - \frac{\sum _{i=1}^{n} ({\textbf {x}}_i - {\textbf {y}}_i)^2}{\sum _{i=1}^{n} ({\textbf {x}}_i - \mu )^2}$	This measures how well the predicted values explain the variance in actual values.

### Segment anything model

Recently, Meta AI developed the segment anything model to identify features in visual data using spatial pattern recognition^58^. It dissects the image in multiple segments using masking which helps in segregating different portions of data. Firstly, a segmentation task specifies an adequate pre-training target with broad array of applications to accommodate adaptable prompts. The model is trained using a comprehensive dataset that includes various variables. In case of limited visual data, an iterative approach is adopted to train the model effectively. It autonomously gathers additional data to enhance the training and reinforce the model using iterations. The prompt may take the form of a single point, bounding box, mask or text input. The prompt based segmentation task can potentially address various downstream segmentation challenges such as zero-shot single-point valid mask evaluation, zero-shot edge detection, zero-shot object proposal and zero-shot instance segmentation. Thus, the output *O* of the model gives a collection of masks using^60^

18 $$\begin{aligned} O = SAM (I * P), \hspace{5mm} \forall P \in \mathscr {P} = \{P_{pt }, P_{bb }, P_{txt }\} \end{aligned}$$

which serve as indicators to the specific item targeted for segmentation. In Eq. 18, *I* and *P* represent the input image and the prompt sampled from the set $\mathscr {P}$, respectively. The subsets $P_{pt }$, $P_{bb }$ and $P_{txt }$ denote point, bounding box and text prompts, respectively. Hence, the SAM integrates three primary components – image encoding, prompt encoding and mask decoding^58^. This helps in rewriting the Eq. 18 as^60^

19 $$\begin{aligned} O = \Phi (\phi (I) * \varphi (P)), \hspace{5mm} \forall P \in \mathscr {P}. \end{aligned}$$

**Fig. 4:**
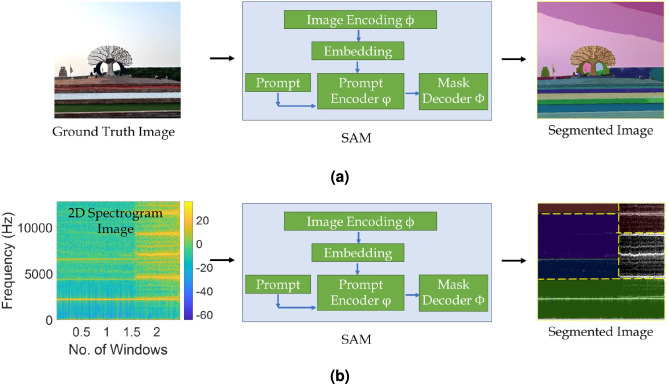
Illustration of the masking process in segment anything model on visual data created using (a) ground truth image and (b) spectrogram of a vibration signal.

The initial step involves utilizing an image encoder $\phi$ to extract the embedding of the input visual data. In addition, a prompt encoder $\varphi$ is utilized to encode the input prompt and obtain the embedding of the prompt. The image and prompt embeddings are entered into a mask decoder $\Phi$ to generate masks under the segmentation process as shown in Fig. 4(a). This indicates a high overlap and pixel-wise agreement between the predicted mask and the ground truth, which demonstrates the effectiveness of SAM in identifying prominent structural boundaries.

**Algorithm 4:**
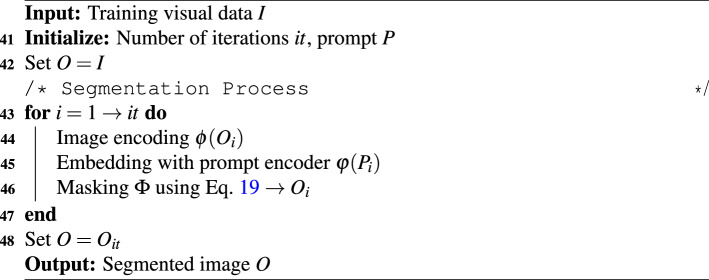
SAM learning framework.

In this study, the image encoder maps a typical $1024\times 1024$ dimension input image to a $64\times 64$ visual data with 256 channels. A pre-trained mask autoencoder with vision transformer is used to divide the input image into multiple batches. Here, 75% of the total batches are masked randomly and the remaining unmasked batches are passed through an encoder vision transformer to the output embeddings^60^. The encoder maps the input mask to $64 \times 64$ dimension with similar channels and adds it to the image embeddings before passing to the mask decoder. In case of encoding point based prompts $P_{pt }$, the positional embedding of the selected point is trained with unique embedding of the foreground and background points in the visual data. It is used to encode the prompted point to identify the false negative or positive error region. The boundary box prompt $P_{bb }$ encoding is trained using the sum of the positional embeddings of the top left and bottom right corners with special embeddings. A pre-trained model (i.e., clip) is used for encoding the text based prompts in the visual data. All prompt token embeddings are arranged in a 2D matrix in the mask decoder $\Phi$. This results in three new output tokens added in the embeddings for each prompt. Subsequently, a two-stage layer is applied to the prompt embeddings, wherein the first layer is a self-attention layer to enrich each embedding for content awareness. In the second layer, these prompts are updated using cross-attention with the image embedding. This introduces more inductive bias to the embeddings and awareness of the original geometrical properties of the actual image^61^. Further, the image embeddings are upsampled to a $512\times 512$ feature space with 256 dimensions. The final prompt embeddings extract three outputs from the corresponding three prompts and pass through multi-layer perceptron (MLP)^62^ to map each prompt with the number of channels (i.e., 256). The SAM model evaluates the intersection over union (IoU) score as^63^

20 $$\begin{aligned} IoU = \frac{|E \cap F|}{|E \cup F|}, \hspace{5mm} \in [0, 1] \end{aligned}$$

which measures the overlap between the original and predicted prompts, where *E* and *F* are two arbitrary shapes. Additionally, the mask prediction is tested for the linear combination of losses such as the dice loss $\mathscr {D}_l$ calculated using^61^

21 $$\begin{aligned} \mathscr {D}_l=\frac{2\sum _{i}^{N} E_i F_i}{\sum _{i}^{N} E_i^2+ \sum _{i}^{N} F_i^2} \end{aligned}$$

and focal loss $\mathscr {F}_l$ determined as^64^

22 $$\begin{aligned} \mathscr {F}_l(\mathscr {T}_t) = - [1-\mathscr {T}_t^{\eta } \ln {(\mathscr {T}_t)} ] \end{aligned}$$

where, $\mathscr {T}_t$ is the probability of prediction class and power term $\eta \in [0,5]$. The SAM also estimates the mean square error (MSE) loss between the prediction and the actual visual data. Performance metrics are determined as illustrated in Table 2, and in addition, the accuracy and the boundary F1 score are also used for this learning model. The accuracy is the ratio of accurate outcomes to total cases and is calculated using $(n_{TP } + n_{TN }) / (n_{TP } + n_{FN } + n_{FP } + n_{TN })$. The boundary F1 score is a measurement of segmentation accuracy along the object edges, which is evaluated as

23 $$\begin{aligned} Boundary F1 score = 2 \times \frac{Precision _{b} \times Recall _{b}}{Precision _{b} + Recall _{b} } \end{aligned}$$

where, the term $Precision _{b}$ denotes the proportion of predicted boundary pixels that are within an acceptable distance of the ground truth boundary pixels. In Eq. 23, $Recall _{b}$ is the proportion of ground truth boundary pixels that match predicted boundary pixels within the allowable tolerance. Fig. 4(b) shows a stepwise procedure of the SAM for visual data learning to create a segmented image and an iterative process is summarized in Algorithm 4.

**Fig. 5:**
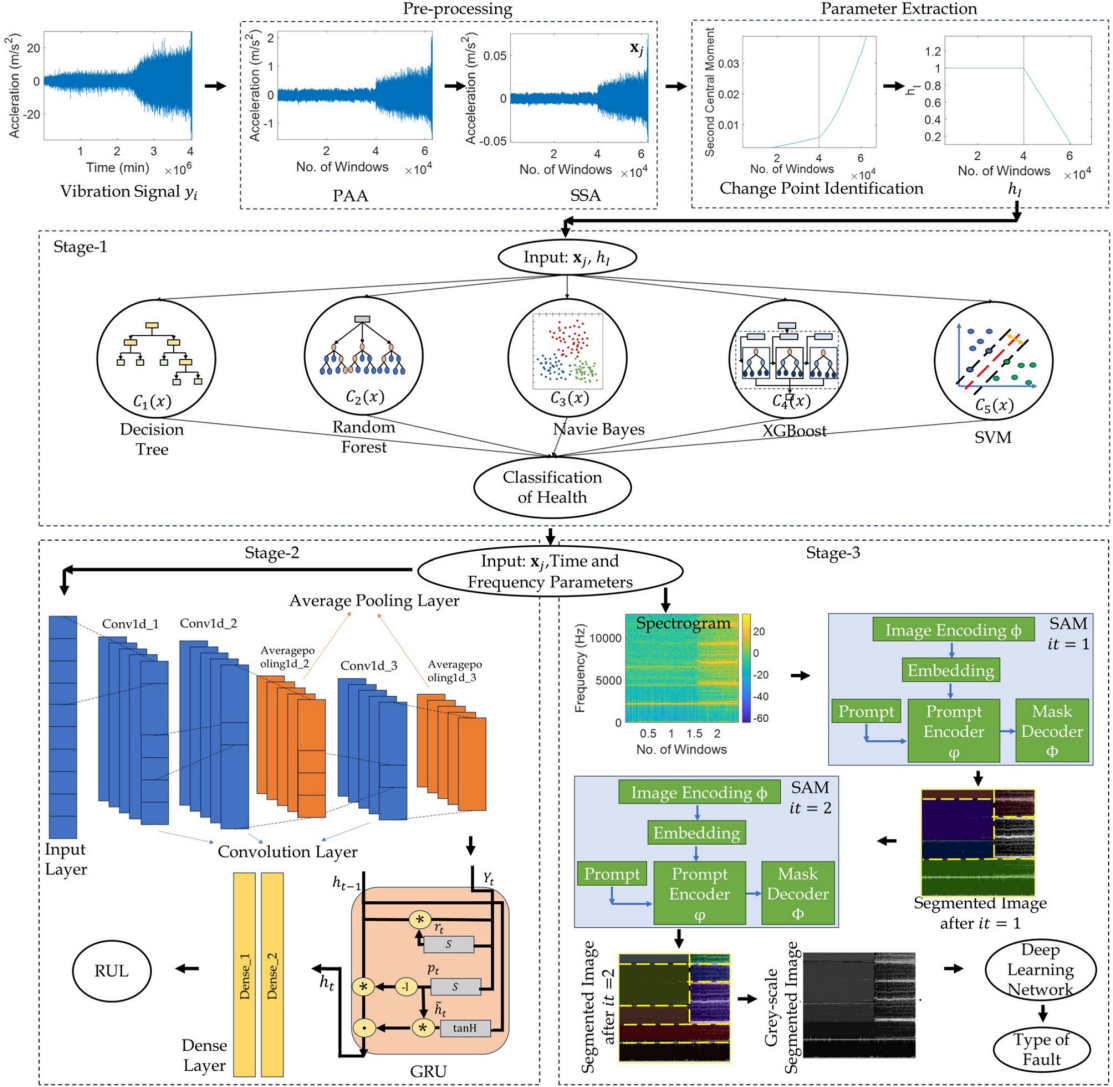
Flowchart of the proposed multi-stage e-CNN-GRU-SAM framework for classification of health, prognostic analysis and identification of fault type.

## Proposed e-CNN-GRU-SAM network

This section explains the proposed network using the earlier discussed voting ensemble, CNN, GRU and segment anything based learning models (i.e. e-CNN-GRU-SAM). In this study, the time series data is streamlined for better efficiency as suggested in pre-processing. At first, the PAA is used to create multiple scaled time series windows using Eq. 1. The noise in the dataset is filtered using SSA (i.e., Eq. 4) without marginalizing the statistical and critical features. Following Algorithm 1, the raw time series signal of the bearings is conditioned in this study. The proposed framework determines the classification of health, the prediction of remaining service life of bearings and the identification of failure type in different stages. These stages require multiple learning tools and their basic architectures are illustrated in the earlier sections. The application of these aforementioned learning models is further explained as:

- *Stage-1 (Classification of Health)*: The first stage of the condition assessment requires detection of the fault in the system. Here, the study proposes voting ensemble based learning (following Eq. 9) as the characterization requires binary classification of health. Different ML based classifiers are considered in this study such as decision tree, random forest, naive Bayes, XGBoost and SVM^56^. The hard voting as per the Algorithm 2 is performed on the prediction of bearing health and the majority result is used to characterize it. The performance of the bearing health classification is assessed using the evaluation metrics as mentioned in the Table 2. The choice of aforementioned classifiers is based on the wide application in the literature^56^ and the present proposal provides no limitation on the selection of the classifiers.
- *Stage-2 (Prediction of RUL)*: In the next stage, the useful service life of the bearing post damage initiation is estimated using the CNN-GRU model as discussed earlier. This deep network of convolution, average pooling and GRU layers is trained (as per the Algorithm 3) using the input time and frequency parameters extracted from the pre-processed time series data. The output is the estimation of the bearing health index $h_I$ from the vibration response. This value helps in the prognostic analysis of the bearings using Eq. 8 to predict RUL. The efficiency of the prediction of the bearing service life is evaluated using performance metrics as presented in the Table 3.
- *Stage-3 (Identification of Type of Fault)*: It is predicted using a novel visual data classification approach based on the SAM and deep network. The time series data is converted into a spectrogram image which facilitates depiction of the spatial-temporal features of the frequency components in a 2D visual data. The short-time Fourier transform (STFT) technique is used to cater non-stationary vibration responses as the data series is partitioned into shorter time intervals and hence, capturing the variation in the frequency characteristics over time. The transformation $\varvec{X}_f(\tau ,\omega )$ of the time series $\varvec{X}$ is expressed as^11,65^,
  24 $$\begin{aligned} \varvec{X}_f(\tau ,\omega ) = \int _{-\infty }^{\infty } \varvec{X}(t)\Gamma (t-\tau )e^{-\iota \omega t} \,dt \end{aligned}$$
  where, $\Gamma (\bullet )$ is a truncated Gaussian function. The magnitude of the frequency is depicted using colour intensity and hence, the visual data is presented as a coloured image. As suggested earlier, the window size in SAM is considered as 512 to maintain consistency. The sample overlap between successive windows is 256 to facilitate seamless transition between consecutive sections of the time signal. A large value can be selected to ensure high frequency resolution of the resulting spectrogram image, albeit increased computational cost. Hence, a trade-off is suggested and the number of time steps equal to 1024 is adopted in this study. This value may be altered as per the frequency of the time series data. In this study, the spectrogram image is used by the SAM to mask the distinct features in the visual data. The masking process may be refined in multiple iterations to mark the changes in the frequency features. Here, two iterations are adopted to mask the images and subsequently, the segmented visual data is normalized to a grey-scale image for ease of learning. It is further used to train deep networks such as ANN, CNN, LeNet5^66^, ResNet50^67^, AlexNet^68^ and GoogLeNet^68^, for prediction of the nature of fault in the bearing. This yields classification of the failure and its performance can be evaluated using the aforementioned evaluation metrics such as precision, recall, accuracy, F1 score and boundary F1 score.

The operational flowchart of the proposed e-CNN-GRU-SAM network for the generalized diagnosis of bearing faults is shown in Fig. 5. Since the classification of an unhealthy bearing (in Stage-1), the prediction of the RUL and the identification of the fault type (i.e., Stage-2 and Stage-3, respectively) are executed simultaneously in this forensic analysis.

**Fig. 6:**
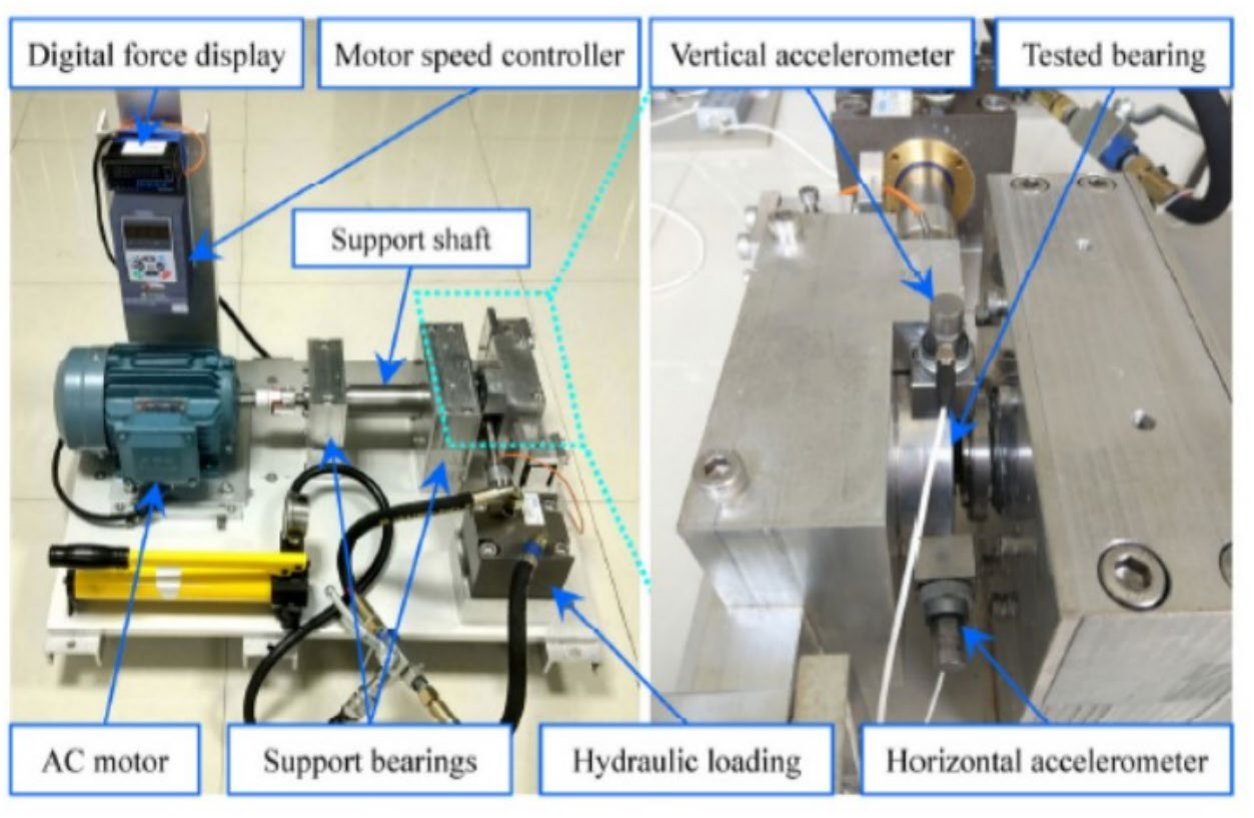
Experimental setup for the fault diagnosis of rolling bearings by XJTU-SY^69^.

**Fig. 7:**
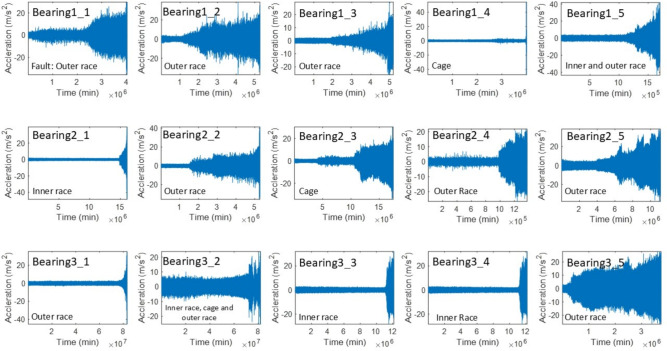
XJTU-SY dataset of the rolling bearing vibration signals till failure.

**Table 4:** Details of operating conditions and failures of the XJTU-SY bearing dataset^69^.

**Operating condition**	**Rotating speed (rpm)**	**Load (kN)**	**Dataset nomenclature**	**Bearing lifetime**	**Fault elements**
1	2,100	12	Bearing1_1	2h 3m	Outer race
			Bearing1_2	2h 41m	Outer race
			Bearing1_3	2h 38m	Outer race
			Bearing1_4	2h 2m	Cage
			Bearing1_5	52m	Inner race and outer race
2	2,250	11	Bearing2_1	8h 11m	Inner race
			Bearing2_2	2h 41m	Outer race
			Bearing2_3	8h 53m	Cage
			Bearing2_4	42m	Outer race
			Bearing2_5	5h 39m	Outer race
3	2,400	10	Bearing3_1	42h 18m	Outer race
			Bearing3_2	41h 36m	Inner race, ball, cage and outer race
			Bearing3_3	6h 11m	Inner race
			Bearing3_4	25h 15m	Inner race
			Bearing3_5	1h 54m	Outer race

**Fig. 8:**
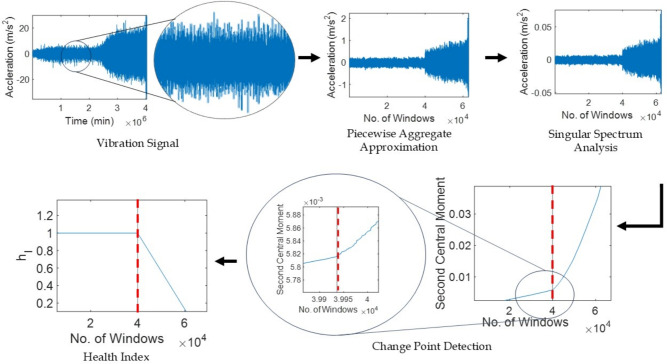
Illustration of the pre-processing of bearing vibration response of Bearing1_1 as per the proposed framework.

## Results and discussion

The proposed e-CNN-GRU-SAM network with pre-processing is applied to assess bearing diagnosis and prognosis. This framework is performed for classification of health, prediction of RUL and determination of fault type. As discussed earlier, a combination of multiple learning networks is proposed to address these challenges in a novel single multi-stage approach. The results are compared with other widely used DL models such as ANN, LSTM, autoencoder and sparse autoencoder^70^ in assessing the classification of health under the Stage-1. Since the models result in binary outputs, the metrics such as precision, recall and F1 score are used to evaluate their respective performances. Furthermore, state-of-the-art DL models such as DANN^40^, TCN-SA^42^, ResNet-MA^41^, GRU-DeepAR^44^, SACGNet^43^, CAMTCN^45^, CARDenseNET^49^ and TCN-transformer^71^ are compared to the Stage-2 of the proposed e-CNN-GRU-SAM network to evaluate the efficacy in RUL prediction. Here, the performance metrics are based on the estimation of error using MAE, RMSE and R2. Finally, the type of bearing failure is identified in the Stage-3 using different deep networks such as ANN, CNN, LeNet5^66^, ResNet50^67^, AlexNet^68^ and GoogLeNet^68^ after SAM based masking of the visual data. The performance metrics include precision, recall, accuracy, F1 score and boundary F1 score as illustrated earlier.

**Fig. 9:**
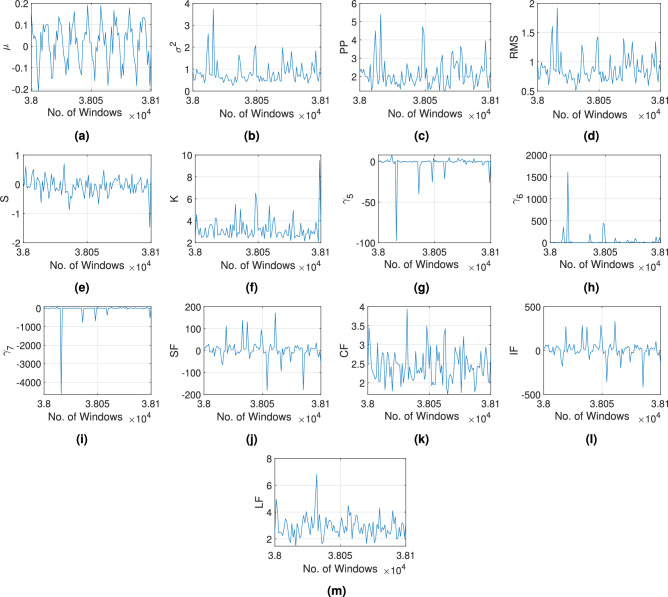
Sensitivity of the time domain parameters such as (**a**) $\mu$, (**b**) $\sigma ^2$, (**c**) PP, (**d**) RMS, (**e**) $\mathscr {S}$, (**f**) $\mathscr {K}$, (**g**) $\gamma _5$, (**h**) $\gamma _6$, (**i**) $\gamma _7$, (**j**) SF, (**k**) CF, (**l**) IF and (**m**) LF, extracted during pre-processing of Bearing1_1 vibration signal from XJTU-SY dataset.

**Fig. 10:**
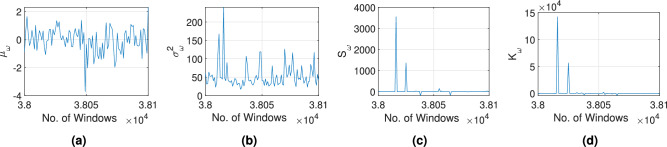
Sensitivity of the frequency domain parameters such as (**a**) $\mu _{\omega }$, (**b**) $\sigma _{\omega }^2$, (**c**) $\mathscr {S}_{\omega }$ and (**d**) $\mathscr {K}_{\omega }$, extracted during pre-processing of Bearing1_1 vibration signal from XJTU-SY dataset.

In this study, two different experimental datasets as suggested by Wang et al. (2018)^69^ and Lessmeier et al. (2016)^72^ are considered to replicate failure scenarios such as wind turbine gearbox bearings in consistent operations^73^. These datasets are widely known as the Xi’an Jiaotong University and Changxing Sumyoung Technology Co., Ltd. (XJTU-SY) bearing data and the Paderborn University (PU) bearing data, respectively. The datasets include the vibration responses of bearings that operate under different conditions and are further discussed in the following sections.

**Fig. 11:**
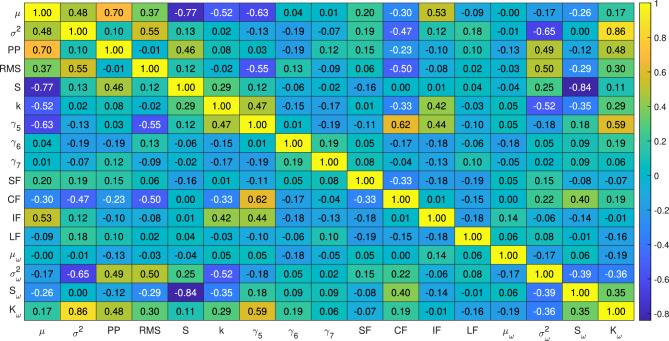
Correlation between the time and frequency parameters extracted from the XJTU-SY dataset.

**Table 5:** Ablation analysis of voting ensemble and CNN-GRU for classification of health and prediction of RUL using XJTU-SY dataset.

**Model/**	**Performance**	**Window Size** *w*					
**Statistical Parameter**	**Measure**	**32**	**64**	**96**	**128**	**192**	**256**
Change point detection	-	Yes	Yes	Yes	Yes	Yes	No
Voting ensemble	F1 score	0.987	0.987	0.819	0.752	0.722	-
	Training time	266.687	105.137	99.049	87.192	67.625	-
CNN	MAE	0.290	0.292	0.566	0.581	0.627	-
	RMSE	0.421	0.377	0.749	0.752	0.811	-
	R2	0.215	0.252	0.170	0.037	0.016	-
	Training time	477.308	155.484	152.254	148.014	112.743	-
GRU	MAE	0.265	0.220	0.408	0.566	0.579	-
	RMSE	0.438	0.327	0.610	0.730	0.800	-
	R2	0.328	0.331	0.226	0.110	0.054	-
	Training time	634.632	209.486	166.678	122.073	130.073	-
Transformer	MAE	0.245	0.248	0.327	0.494	0.577	-
	RMSE	0.286	0.287	0.403	0.621	0.782	-
	R2	0.365	0.363	0.261	0.221	0.118	-
	Training time	480.939	316.740	257.626	219.953	201.841	-
CNN-Transformer	MAE	0.068	0.049	0.152	0.217	0.288	-
	RMSE	0.072	0.066	0.190	0.286	0.319	-
	R2	0.864	0.925	0.560	0.361	0.392	-
	Training time	386.160	255.768	228.493	200.328	187.116	-
CNN-GRU	MAE	0.050	0.007	0.108	0.120	0.187	-
	RMSE	0.064	0.009	0.243	0.260	0.355	-
	R2	0.954	0.999	0.530	0.496	0.401	-
	Training time	208.739	70.860	69.569	66.539	60.692	-

**Table 6:** Ablation experiment of IoU thresholds for segmentation performance in masking iterations using XJTU-SY dataset.

**IoU Score**	**Masking **$it=1$					**Masking **$it=2$				
	**F1 Score**	**Accuracy**	**Boundary F1 Score**	**Precision**	**Recall**	**F1 Score**	**Accuracy**	**Boundary F1 Score**	**Precision**	**Recall**
0.60	0.8742	0.9764	0.7758	0.8854	0.8632	0.8776	0.8565	0.7725	0.8872	0.8682
0.65	0.8860	0.9785	0.7914	0.8926	0.8795	0.8869	0.8583	0.7960	0.8943	0.8796
0.70	0.9061	0.9814	0.8107	0.9181	0.8945	0.8957	0.8607	0.8058	0.9092	0.8825
0.75	0.9375	0.9815	0.8831	0.9504	0.9245	0.9107	0.8616	0.8230	0.9204	0.9011
0.80	0.9016	0.9722	0.8556	0.9129	0.8913	0.9232	0.8729	0.8248	0.9301	0.9165
0.85	0.8783	0.9721	0.8176	0.9015	0.8588	0.9843	0.8892	0.9643	0.9332	0.9843
0.90	0.8642	0.9705	0.8029	0.8961	0.8433	0.9419	0.8840	0.9129	0.9314	0.9527

**Table 7:** Optimized hyperparameters of the classifiers in the Stage-1 using the GridSearchCV method.

**Classifiers**	**Hyperparameter**	**Optimized Hyperparameter Value**
Decision tree	Maximum depth [3, 5, 7]	7
	Minimum samples split [2, 4, 6]	6
	Criterion [Gini, entropy]	Entropy
Random forest	Maximum depth [3, 5, 7]	7
	Minimum samples split [2, 4, 6]	6
	Number of estimators [50, 100, 200]	100
	Maximum features [square root, logarithmic]	Square root
Naive Bayes	Variance smoothing [logspace(1 to 1E$-9$)]	8.11E$-5$
XGBoost	Maximum depth [3, 5, 7]	7
	Learning rate [0.01, 0.10, 0.50]	0.10
	Number of estimators [50, 100, 200]	200
	Minimum child weight [1, 3, 5]	3
	Gamma [0, 0.1, 0.5]	0.5
SVM	C [0.1, 1, 10]	1
	Kernel [linear, RBF, polynomial]	RBF
	Gamma [scale, auto]	Scale

**Table 8:** F1 score and training time of different ML models for classification of the bearing health using the XJTU-SY dataset.

Dataset	Proposed Network	ANN	LSTM	Autoencoder	Sparse Autoencoder
Bearing1_1	0.9723	0.9426	0.9735	0.9738	0.8638
Bearing1_2	0.9701	0.9121	0.9606	0.8864	0.9355
Bearing1_3	0.9911	0.9653	0.9748	0.9458	0.9208
Bearing1_4	0.9978	0.9638	0.9689	0.9383	0.9483
Bearing1_5	0.9960	0.9496	0.9511	0.9469	0.9469
Bearing2_1	0.9928	0.9783	0.9773	0.9659	0.9716
Bearing2_2	0.9774	0.9459	0.9782	0.9651	0.9428
Bearing2_3	0.9806	0.9311	0.9836	0.9580	0.9059
Bearing2_4	0.9951	0.9877	0.9873	0.9706	0.9706
Bearing2_5	0.9865	0.9637	0.9878	0.7719	0.8239
Bearing3_1	0.9748	0.9281	0.9651	0.9368	0.9411
Bearing3_2	0.9904	0.9766	0.9680	0.9750	0.9723
Bearing3_3	0.9984	0.9683	0.9799	0.9658	0.9658
Bearing3_4	0.9938	0.9011	0.8688	0.8688	0.9072
Bearing3_5	0.9924	0.9466	0.9847	0.9627	0.9416
Average F1 Score	**0.9873**	**0.9493**	**0.9755**	**0.9355**	**0.9305**
Training Time (s)	**105.1370**	**672.8277**	**1038.5275**	**1477.1289**	**1438.2094**

### Experiment 1: XJTU-SY dataset

The experimental setup of the XJTU-SY dataset shown in Fig. 6 consists of the shaft supported by bearings (as a drivetrain), an AC motor with a speed controller for rotational motion and hydraulic load to apply forces on the shaft^69^. The vibrational responses of the bearings as shown in Fig. 7 are recorded using accelerometers (PCB ICP^®^352C33) at a sampling frequency of 25.6 kHz (https://drive.google.com/open?id=1_ycmG46PARiykt82ShfnFfyQsaXv3_VK). In total, this dataset offers 15 cases of rolling bearings in which vibration responses are recorded until failure. The operating conditions are varied in the rotation speeds and the load applied on the shaft as mentioned in Table 4. The bearings suffer failures predominately in the outer race, inner race and cage in this dataset. It can be noted that the number of cases for each failure type is limited, unbalanced and mixed here. Thus, this presents an interesting challenge for the proposed framework to establish its potential for generalized application.

#### Sensitivity and ablation analysis

Raw vibration signals are pre-processed using PAA and SSA according to the Algorithm 1. The time and frequency domain parameters are extracted according to Table 1 and the change point is detected based on the second central moment using Eq. 7. The threshold of the change point $c_{th}$ is selected using a maximum value of six. Subsequently, the bearing health index $h_I$ is evaluated following Eq. 8. A typical example of the aforementioned pre-processing of the Bearing1_1 vibration response is illustrated in Fig. 8. The statistical features of the time and frequency domains are calculated from the processed signal data presented in Figs. 9 and 10, respectively. The sensitivity of these time and frequency features is evaluated using a linear correlation as shown in Fig. 11. Parameters with correlation factor $< 0.20$ are considered insignificant in this study. Thus, features with a lower correlation magnitude with all the other parameters are screened for better efficiency. Subsequently, the features such as $\gamma _6$, $\gamma _7$, LF and $\mu _{\omega }$ are curtailed for further usage in the training. In concise, 10 time domain and three frequency domain features are found to be significant and are used to train the ML models. The dataset is used to train different models under Stage-1, Stage-2 and Stage-3 for bearing condition assessment after pre-processing and estimation of the features.

Furthermore, ablation experiments are performed to study the role of the proposed e-CNN-GRU-SAM network in fault diagnosis and prognosis. It includes parameters such as window size *w* used in the pre-processing of vibration signals, training of the ensemble voting for classification of health (i.e. Stage-1), performance of CNN and GRU models for prediction of RUL of bearings (i.e. Stage-2), and effect of IoU score in segmentation (i.e. Stage-3). This aims to identify the efficient parameters used in the proposed e-CNN-GRU-SAM network, as well as to signify their critical roles in improving prediction accuracy and reducing computational cost. The ablation study presented in Table 5 highlights the choice of window size and the improvements in learning of the CNN-GRU based network proposed for RUL. In Stage-1 assessment, change point detection is required to define health regimes (i.e., healthy or unhealthy). The result demonstrates higher *w* value leads to faster training of ML classifiers, but with reduced accuracy in terms of the F1 score. An increased window size value *w* also results in unidentified change points. Hence, selecting an optimal window size *w* can enhance the accuracy of the model, as well as computational efficiency. In RUL analysis (under Stage-2), the time required to individually train CNN, GRU and transformer learning^71^ is higher than the proposed combined CNN-GRU architecture due to the deep layering of the learning network. It is observed that the transformer learning model yields less error than the individual CNN and GRU models in RUL prediction. However, their combinations with CNN result in better performance, where the proposed combination of CNN and GRU significantly improves in predicting the RUL with a training time of approx. 70 s. Based on the ablation study, an adequate window size *w* of 64 is adopted in this study considering an insignificant loss in statistical information of the raw vibration data. It can be noted that the window size *w* may vary depending on the vibration data and the time efficiency. Table 6 illustrates the ablation study related to the intersection measure of segmentation in Stage-3. It is performed for both iterations of the SAM where different values of the IoU score are adopted to determine the performance metrics such as precision, recall, accuracy, F1 score and boundary F1 score. In the first iteration (i.e., $it = 1$), the performance metrics suggest that the IoU score of 0.75 yields a better result and hence, the same was adopted in this study. However, a different IoU score reflects better performance in the second iteration (i.e., $it = 2$) as presented in Table 6. In this iteration step, the IoU score is changed to 0.86^74^ for further applications in this study.

**Fig. 12:**
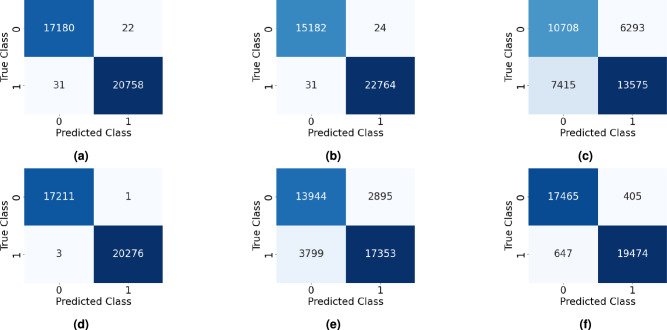
Confusion matrices of the Bearing1_1 XJTU-SY dataset for different ML classifiers from (**a–e**) used in the study [such as decision tree, random forest, naive Bayes, XBGoost and SVM, respectively] and finally, (**f**) the voting ensemble model.

#### Stage-1: Classification of bearing health

In the first stage, ML models are trained to classify bearings as either healthy or unhealthy. This binary classification of the bearing health is essential due to the occurrence of the change point which initiates the degradation process. It assists in mitigation of computational expenses over redundant time (i.e. healthy state) and serves as prompter for the early warning of fault in the system. In the proposed study, this classification problem is addressed by the voting ensemble learning model with ML classifiers – decision tree, random forest, naive Bayes, XGBoost and SVM^56^. In case of the decision tree model, the hyperparameter maximum depth is considered as 3, 5 and 7 with minimum sample split of 2, 4 and 6. These hyperparameters use the Gini and entropy criteria to train the model. Similar values of the maximum depth and minimum sample split are considered for the random forest model. It requires additional parameters such as the number of estimators (i.e., 50, 100 and 200) and the maximum features (i.e., square root and logarithmic). The naive Bayes classifier uses a single hyperparameter with array ranging between $[-9,$ 0] required for the variance smoothing. It generates an array of 100 logarithmically spaced values of the parameter ranging from 1 to 1E$-9$ which is optimized during the hyperparameter tuning. The XGBoost classifier uses the hyperparameters such as the maximum depth (i.e., 3, 5 and 7), learning rate (say 0.01, 0.10 and 0.50), number of estimators (i.e., 50, 100 and 200), the minimal sum of instance weight (Hessian) required in a child (i.e., 1, 3 and 5) and the regularization parameter gamma (i.e., 0, 0.1 and 0.5). In SVM, the regularization parameter to govern the balance between maximizing margin and minimizing classification errors is selected among values 0.1, 1.0 and 10.0. In this study, the performance of three distinct kernel functions, *viz.* linear, RBF and polynomial (2$^{nd }$ order), are considered and the parameter gamma (i.e., scale and auto) is used to determine the shape of the decision border. The optimized hyperparameters are determined using the grid search cross-validation (GridSearchCV) method^56^ as presented in the Table 7 and the same are used in the training of the classifiers. The training is performed using the first dataset for a given operating condition of the bearing, i.e. Bearing1_1, Bearing2_1 and Bearing3_1, and the rest are used for testing the models. Fig. 12 shows the confusion matrices from the aforementioned classifiers and voting ensemble model using the Bearing1_1 vibration dataset. The confusion matrices indicate the number of TP, FP, TN and FN instances after the training and the same process is repeated on the other bearing data. The F1 score of the voting ensemble is compared with DL models such as ANN, LSTM, autoencoder and sparse autoencoder^70^, to assess their performance as presented in Table 8. It can be observed that the proposed voting ensemble provides improved F1 score and training time than ANN, LSTM, autoencoder and sparse autoencoder in classifying the health of the bearings used in this study. The performance is enhanced using the ensemble model which requires hard voting from different classifiers and thus, the average F1 score is approx. 0.987 considering different working conditions with a training time of about 105 s.

**Table 9:** The proposed CNN-GRU architecture in the Stage-2 of the present framework to predict the bearing RUL.

**Layer Name**	**Layer Type**	**Activation Size **(**Spatial **$\times$ **Channels **$\times$ **Batch**)	**Learnable Parameters**
Conv1d_1	Convolution	13$\times$256$\times$1	Weight: (3,1,256)
			Bias: (256,)
			Parameters: 1024
ReLU_1	Activation Function	13$\times$256$\times$1	-
Conv1d_2	Convolution	11$\times$128$\times$1	Weight: (3,256,128)
			Bias: (128,)
			Parameters: 98432
ReLU_2	Activation Function	11$\times$128$\times$1	-
Averagepooling1d_2	Average pooling layer	5$\times$128$\times$1	-
Conv1d_3	Convolution	3$\times$128$\times$1	Weight: (3,128,32)
			Bias: (32,)
			Parameters: 12320
ReLU_3	Activation Function	1$\times$32$\times$1	-
Averagepooling1d_3	Average pooling layer	1$\times$32$\times$1	-
GRU	GRU	1$\times$32$\times$1	Weight: (128,96)
			Recurrent: (32,96)
			Bias: (96,)
			Parameters: 6336
ReLU_4	Activation Function	1$\times$32$\times$1	-
Flatten	Flatten	1$\times$32$\times$1	-
Dense_1	Dense	1$\times$128$\times$1	Weight: (32,128)
			Bias: (128,)
			Parameters: 4224
ReLU_dense_1	Activation Function	1$\times$128$\times$1	-
Dense_2	Dense	1$\times$1$\times$1	Weight: (128,1)
			Bias: (1,)
			Parameters:129

**Table 10:** Comparison of the performance metrics in predicting RUL of XJTU-SY bearings.

**Models**	**RMSE**	**MAE**	**R2 score**
DANN^40^	23.50	26.82	-
TCN-SA^42^	15.77	19.43	-
ResNet-MA^41^	2.49	2.46	-
GRU-DeepAR^44^	0.35	0.29	-
SACGNet^43^	0.30	0.23	-
CAMTCN^45^	12.40	9.66	-
CARDenseNet^49^	0.04	0.05	0.97
DiIncepNet^48^	0.03	0.06	0.79
TCN-transformer^71^	0.05	0.04	0.95
Proposed Network	0.01	0.01	0.99

**Fig. 13:**
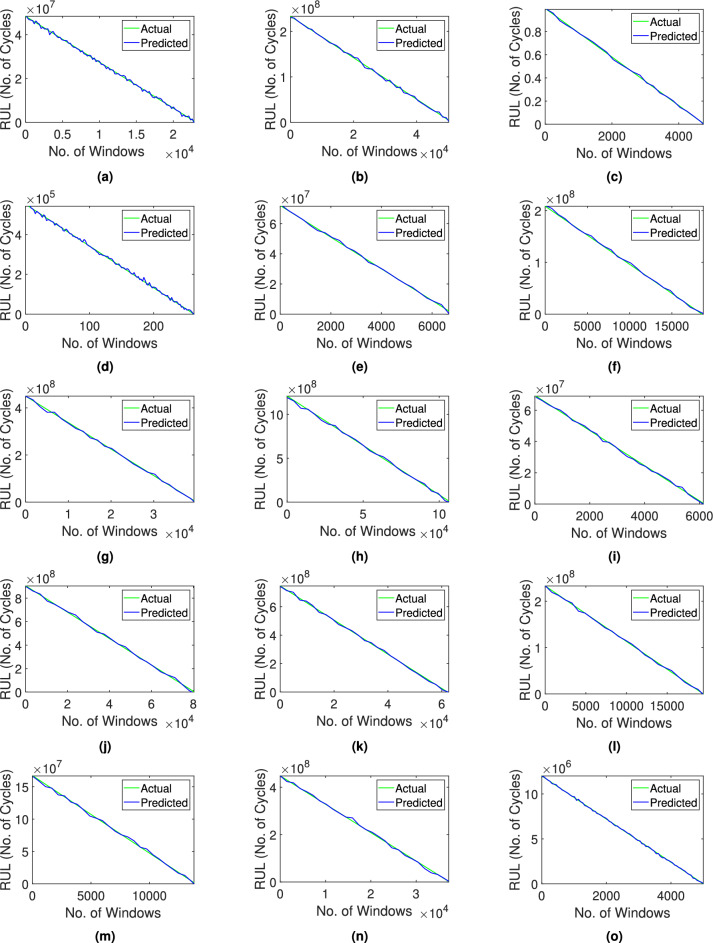
Prediction of the remaining service life of (**a-e**) Bearing1 operating with 2,100 rpm and 12 kN, (**f-j**) Bearing2 operating with 2,250 rpm and 11 kN, and (**k-o**) Bearing3 operating with 2,400 rpm and 10 kN, using the proposed network.

#### Stage-2: Prediction of RUL

Once the bearing is classified as unhealthy in the Stage-1, the remaining serviceable life of the system is predicted in this stage. It requires significant time and frequency parameters to train the proposed CNN-GRU network. The architecture of this DL model consists of three convolution layers, two average pooling layers, a GRU and a flatten layer followed by two dense layers, as summarized in Table 9. In the convolution layers, the kernel size is considered as $3\times 3$, which can be changed as defined by the user. The initial layer reduces the dimension from 13 to 11 and in the subsequent layers, the dimension is further curtailed to 3. These layers employ a convolution process as described in Eq. 10 to extract significant characteristics from the input data. An average pooling layer follows each convolutional layer to aid in the reduction of the dimension due to the filtering of crucial information. The GRU layer is a variant of the RNN architecture designed to effectively model sequential patterns and relationships within the given input data. The respective output is compressed into the form of $1\times 32$ using the architecture as given in Table 9. The flatten layer transforms the input data into a 1D vector which is further used by the two layers of high neuron density. The inclusion of these layers introduces non-linearity into the model for better learning of the input-output relation. Overall, it culminates in a singular scalar output through the last dense layer. In this study, the ReLU activation function with a batch size of 32 and 100 epochs is used. Optimization is performed using an adaptive moment estimation (ADAM) technique^70^ based on an iterative stochastic gradient descent framework. The learning rate of the optimization process is selected as 0.01 based on accuracy and efficiency. As suggested earlier, different state-of-the-art networks such as DANN^40^, TCN-SA^42^, ResNet-MA^41^, GRU-DeepAR^44^, SACGNet^43^, CAMTCN^45^, DiIncepNet^48^, CARDenseNET^49^ and TCN-transformer^71^ are compared with the given data for the prediction of bearing RUL. Similar to the Stage-1, the aforementioned models are trained with a single dataset of a given bearing operating condition and the remaining datasets are used for testing purposes. The performance of these models are measured using MAE, RMSE and R2 metrics. Fig. 13 shows the prediction of the service life using the proposed method for each bearings in the XJTU-SY dataset. It can be observed from Table 10 that the proposed network provides a fairly accurate prediction of the RUL of bearings compared to the other DL networks considered in this study with average values of MAE, RMSE and R2 of 0.01, 0.01 and 0.99, respectively. The proposed framework effectively incorporates pre-processing to enable denoising and compression of the vibration signals while preserving essential statistical properties. This transformation maintains the fundamental temporal patterns and facilitates the extraction of time- and frequency-domain properties. Consequently, the network is trained on high-quality, noise-resistant data representations, hence enhancing the accuracy of the trained model for bearing prognosis under different failure scenarios. The results clearly illustrate the improvements in the accuracy and efficiency using the proposed framework.

#### Stage-3: Identification of type of fault

**Fig. 14:**
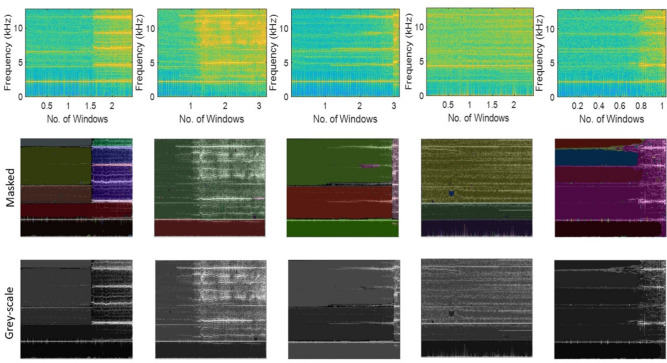
Masking of the spectrogram images using SAM and grey-scaling of the vibration response of XJTU-SY bearings [i.e., Bearing1_1, Bearing1_2, Bearing1_3, Bearing1_4 and Bearing1_5 (from left to right)] operating under the condition 1.

**Fig. 15:**
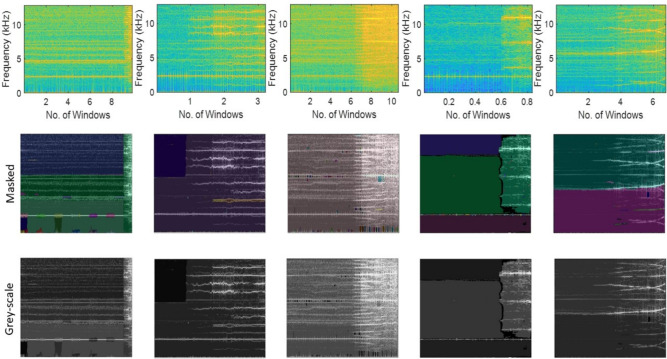
Masking of the spectrogram images using SAM and grey-scaling of the vibration response of XJTU-SY bearings [i.e., Bearing2_1, Bearing2_2, Bearing2_3, Bearing2_4 and Bearing2_5 (from left to right)] operating under the condition 2.

**Fig. 16:**
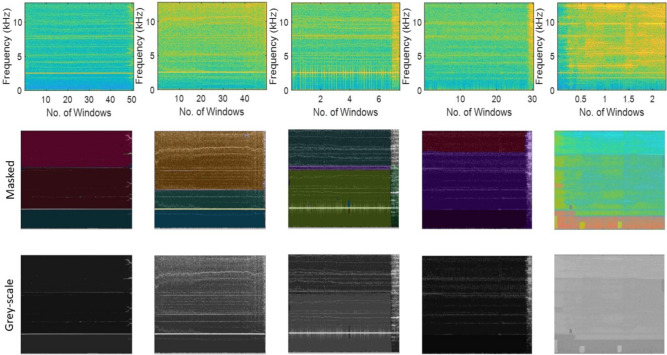
Masking of the spectrogram images using SAM and grey-scaling of the vibration response of XJTU-SY bearings [i.e., Bearing3_1, Bearing3_2, Bearing3_3, Bearing3_4 and Bearing3_5 (from left to right)] operating under the condition 3.

This stage is performed in parallel with Stage-2 after identifying the unhealthy state of the system. As mentioned earlier, this stage uses visual data to train the image processing model (i.e. SAM) to identify the type of failure. The bearing vibration signal is non-stationary as the response changes significantly after the occurrence of damage. Hence, the pre-processed vibration signal is transformed using Eq. 24 to obtain the frequency variation over time. This, in turn, transforms the original vibration data into a 2D spectrogram image of the bearing vibration, where the magnitude of the frequency is depicted using colour intensity. The SAM uses the spectrogram image to perform the image segmenting or masking process as shown in Fig. 14. This process can be performed multiple times for segment refinement as suggested earlier. In the present study, two iterations of the image segmenting process are adopted but are not restrictive and can be defined by the user to enhance the number of masks. The first iteration of image masking in the SAM specifies the number of points or landmarks to be produced on either side of the object. Here, this value is configured to 32 and a threshold IoU value of 0.75 is used to evaluate the accuracy of generated masks as discussed earlier. The masks with IoU score above the threshold value are accepted. A threshold stability score of 0.89 is considered for consistent generation of masks by making it less sensitive to changes^74^. In each processing step, layer cropping or downsampling technique is applied to reduce the resolution of the input image and masks. This helps in mitigating the high density of points in regions of low resolution. Here, the downsampling process is performed with a factor of two during the cropping stage. In addition, regions with area <100 units are eliminated for efficiency. In the second iteration of masking, the threshold values for IoU and stability are assigned as 0.86 and 0.92, respectively, for further refinement. After the iteration of image masking, the visual data is normalized by converting to a grey-scale image. This visual data with segmented features and normalization of the colour intensity is used to train the DL model for the prediction of the fault type. As mentioned earlier, the present study considers a total of 15 cases in the XJTU-SY dataset with primarily three types of damage, *viz.* inner race, outer race and cage. Similarly to Bearing1, the vibration signals of the other bearings (i.e., Bearing2 and Bearing3) are also transformed to masked visual data using STFT and SAM as shown in Figs. 15 and 16, respectively. Table 11 presents the performance evaluation metrics such as precision, recall, accuracy, F1 score and boundary F1 score of the spectrogram segmentation for all bearing cases. In the second iteration, a consistent improvement in the boundary accuracy and overall segmentation metrics is observed from the previous step. The average values are observed as 0.96, 0.92, 0.98, 0.94 and 0.88, respectively, after the second iteration of masking. This indicates the effectiveness of the iterative masking refinement adopted in this proposed network.

**Table 11:** Estimation of segmentation evaluation metrics across masking iterations for the Stage-3 of the proposed network using XJTU-SY dataset.

**Ground**	**Masking **$it=1$					**Masking **$it=2$				
**Truth (Dataset)**	**F1 Score**	**Accuracy**	**Boundary F1 Score**	**Precision**	**Recall**	**F1 Score**	**Accuracy**	**Boundary F1 Score**	**Precision**	**Recall**
Bearing1_1	0.9375	0.9815	0.8831	0.9504	0.9245	0.9487	0.9843	0.8892	0.9643	0.9332
Bearing1_2	0.9210	0.9751	0.8737	0.9412	0.9011	0.9324	0.9773	0.8743	0.9531	0.9127
Bearing1_3	0.9113	0.9728	0.8549	0.9304	0.8920	0.9284	0.9735	0.8614	0.9507	0.9064
Bearing1_4	0.9294	0.9786	0.8775	0.9451	0.9144	0.9439	0.9841	0.8812	0.9654	0.9233
Bearing1_5	0.9418	0.9831	0.8865	0.9586	0.9252	0.9513	0.9876	0.8941	0.9681	0.9346
Bearing2_1	0.9204	0.9716	0.8683	0.9374	0.9024	0.9298	0.9785	0.8755	0.9482	0.9117
Bearing2_2	0.9113	0.9642	0.8542	0.9281	0.8945	0.9246	0.9701	0.8658	0.9361	0.9138
Bearing2_3	0.9254	0.9772	0.8747	0.9441	0.9071	0.9282	0.9749	0.8810	0.9442	0.9131
Bearing2_4	0.9371	0.9827	0.8858	0.9566	0.9182	0.9473	0.9844	0.8922	0.9655	0.9297
Bearing2_5	0.9448	0.9863	0.8918	0.9614	0.9284	0.9571	0.9894	0.8974	0.9809	0.9346
Bearing3_1	0.9093	0.9701	0.8516	0.9274	0.8921	0.9194	0.9727	0.8597	0.9379	0.9009
Bearing3_2	0.9239	0.9754	0.8689	0.9424	0.9057	0.9322	0.9784	0.8754	0.9524	0.9135
Bearing3_3	0.9341	0.9811	0.8850	0.9541	0.9154	0.9439	0.9890	0.8896	0.9620	0.9258
Bearing3_4	0.9471	0.9884	0.8921	0.9631	0.9324	0.9570	0.9921	0.9015	0.9744	0.9440
Bearing3_5	0.9472	0.9849	0.8934	0.9644	0.9301	0.9594	0.9954	0.9172	0.9762	0.9425

**Fig. 17:**
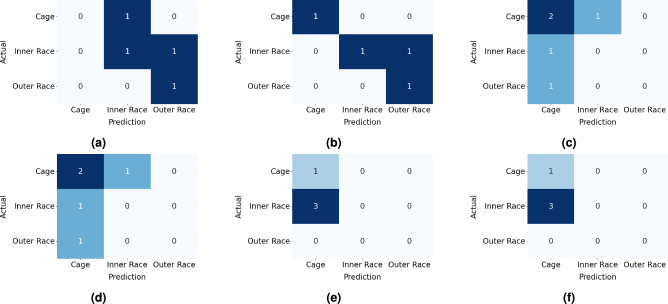
Confusion matrices for prediction of fault types in XJTU-SY dataset using (**a**) SAM-ANN, (**b**) SAM-CNN, (**c**) SAM-LeNet5, (**d**) SAM-ResNet50, (**e**) SAM-AlexNet, and (**f**) SAM-GoogLeNet.

**Table 12:** Outcome of evaluation indices for prediction of fault types in XJTU-SY dataset using SAM with different DL networks.

**Model**	**Fault Type**	**Masking ** $it=1$			**Masking ** $it=2$		
		**Precision**	**Recall**	**F1 Score**	**Precision**	**Recall**	**F1 Score**
SAM-ANN	Outer Race	0.33	0.67	0.40	0.50	1.00	0.67
	Inner Race	0.00	0.00	0.00	0.50	0.50	0.50
	Cage	0.00	0.00	0.00	0.00	0.00	0.00
SAM-CNN	Outer Race	0.33	0.67	0.40	1.00	1.00	1.00
	Inner Race	0.00	0.00	0.00	1.00	0.50	0.67
	Cage	0.00	0.00	0.00	0.50	1.00	0.67
SAM-LeNet5	Outer Race	0.00	0.00	0.00	0.00	0.00	0.00
	Inner Race	0.00	0.00	0.00	0.00	0.00	0.00
	Cage	0.50	0.67	0.57	0.50	0.67	0.57
SAM-ResNet50	Outer Race	0.00	0.00	0.00	0.00	0.00	0.00
	Inner Race	0.00	0.00	0.00	0.00	0.00	0.00
	Cage	0.50	0.67	0.57	0.50	0.67	0.57
SAM-AlexNet	Outer Race	0.00	0.00	0.00	0.00	0.00	0.00
	Inner Race	0.00	0.00	0.00	0.00	0.00	0.00
	Cage	0.25	1.00	0.40	0.25	1.00	0.40
SAM-GoogLeNet	Outer Race	0.00	0.00	0.00	0.00	0.00	0.00
	Inner Race	0.00	0.00	0.00	0.00	0.00	0.00
	Cage	0.25	1.00	0.40	0.25	1.00	0.40

Further, DL models are used in this stage to predict the fault type from the masked visual data. The DL models considered in this study are ANN, CNN, LeNet5^66^, ResNet50^67^, AlexNet^68^ and GoogLeNet^68^ as shown in Table 12. The architecture of the ANN model follows interconnected sequential structure, wherein the first flattened layer has 49,152 elements to transform the multi-dimension visual data input into a vector. It is followed by a dense layer consisting of 128 neurons. In this study, a total of 6,291,584 trainable parameters are used by ANN to effectively capture detailed patterns within the input dataset. Such large learning models often suffer overfitting and to address this issue, a dropout layer is assigned with rate equal to 0.5. This layer randomly deactivates neurons during the training process to enhance the accuracy of the model. Similar to Stage-1, the last layer has a dense structure with 129 training parameters and a single neuron output as it results in the classification of damage type in the bearing. The architecture of CNN consists of a 2D convolutional layer with 32 filters to produce an output of dimension (None, 126, 126, 32). The layer dimension signify the presence of 32 feature maps of each $126\times 126$ pixels. A subsequent maxpooling layer results in a decrease of the spatial dimension (None, 63, 63, 32) of the feature maps. An additional layer of 2D convolution consisting of 64 filters is added in the CNN architecture to improve learning with more detailed features. This results in generation of the feature maps with the dimension of (None, 61, 61, 64). A second maxpooling layer helps in reducing the dimension to (None, 30, 30, 64) which is followed by a flatten layer to transform the 2D feature maps into a 1D vector of 57,600 elements. It is further trained with two completely connected dense layers to get the nature of bearing fault. Here, ReLU is used as an activation function in the hidden layer whereas the sigmoid activation function $\mathscr {S}$ is adopted in the output layer. The architecture for other DL models such as LeNet5, ResNet50, AlexNet and GoogLeNet are adopted as per the literature^66–68^. Based on the datasets and failures, 11 datasets are used to train the DL model and the rest are considered for validation. These models are compared on the basis of precision, recall and F1 score. Table 12 evaluates the performance of these DL models based on masking iterations (i.e. $it=$ 1 and 2). It observes that ANN, CNN, LeNet5 and ResNet50 improve the prediction metrics with refinement of the segments in the second iteration, whereas ResNet50 and GoogLeNet show indifferent performance. After the two iterations, CNN provides a better performance in identification of all the aforementioned type of failures as compared to ANN, LeNet5, ResNet50, AlexNet and GoogLeNet. Similar observations can be presented through Fig. 17 which shows the confusion matrices of different learning networks used in the study. The confusion matrices demonstrate the inability of the ANN, LeNet5, ResNet50, AlexNet and GoogLeNet to identify the types of fault possibly due to limited data. These models are mostly designed for natural image datasets^66–68^ and hence, fail to capture the subtle fragmented spectral patterns that characterize incipient or overlapping fault conditions. This can be attributed to the limited adaptability of the architectures to the spectrogram images, particularly in the models such as LeNet5, ResNet50, AlexNet and GoogLeNet, which needs to be improved. Here, the proposed CNN-based learning architecture proves to be adaptive in predicting the type of faults with limited data inputs. The image classification using CNN with masking of two iterations has an overall accuracy of 75% for the given dataset. This illustrates the potential of the masked visual data based prediction of fault types in bearings using the novel SAM-CNN. It can be noted that the dataset considered here has limited and imbalanced cases of fault type where a few cases show mixed failures. This can potentially limit the performance of the learning networks and hence, the next dataset is considered to address this issue.

**Fig. 18:**
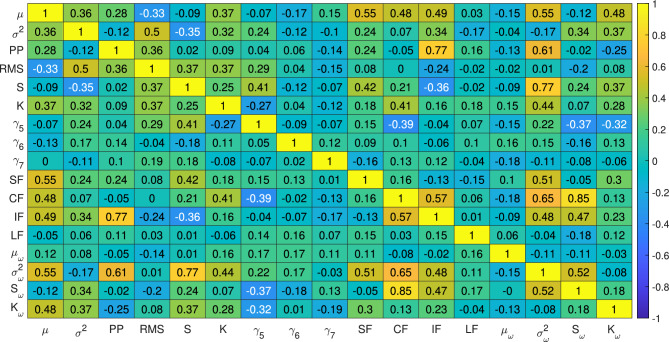
Correlation between the time and frequency parameters extracted from the PU dataset.

**Table 13:** Ablation analysis of voting ensemble and CNN-GRU for classification of health and prediction of RUL using PU dataset

**Model/**	**Performance**	**Window Size** *w*					
**Statistical Parameter**	**Measure**	**32**	**64**	**96**	**128**	**192**	**256**
Change point detection	-	Yes	Yes	Yes	Yes	Yes	No
Voting ensemble	F1 score	0.985	0.985	0.811	0.745	0.710	-
	Training time	315.871	165.336	125.227	102.343	80.657	-
CNN	MAE	0.450	0.462	0.688	0.742	0.751	-
	RMSE	0.543	0.548	0.862	0.877	0.829	-
	R2	0.183	0.161	0.024	0.018	0.005	-
	Training time	513.247	200.914	1194.254	190.615	187.439	-
GRU	MAE	0.442	0.446	0.653	0.704	0.721	-
	RMSE	0.551	0.547	0.821	0.854	0.801	-
	R2	0.163	0.158	0.031	0.024	0.016	-
	Training time	689.114	221.458	215.447	197.615	191.331	-
Transformer	MAE	0.408	0.349	0.527	0.688	0.694	-
	RMSE	0.427	0.486	0.611	0.803	0.872	-
	R2	0.191	0.203	0.174	0.037	0.33	-
	Training time	762.438	428.661	397.126	212.844	210.837	-
CNN-Transformer	MAE	0.07	0.044	0.161	0.209	0.281	-
	RMSE	0.080	0.053	0.212	0.253	0.286	-
	R2	0.881	0.949	0.629	0.680	0.747	-
	Training time	726.159	413.018	384.372	198.770	187.831	-
CNN-GRU	MAE	0.034	0.035	0.112	0.134	0.182	-
	RMSE	0.040	0.045	0.264	0.287	0.390	-
	R2	0.987	0.977	0.504	0.462	0.406	-
	Training time	267.441	71.050	70.546	68.225	62.747	-

**Table 14:** F1 score and training time of different ML models for classification of the bearing health using the PU dataset.

Dataset	Proposed Network	ANN	LSTM	Autoencoder	Sparse Autoencoder
KA01	0.9849	0.8564	0.9642	0.9235	0.9848
KA03	0.9661	0.9219	0.9317	0.9527	0.9794
KA04	0.9939	0.9036	0.9544	0.9303	0.9366
KA05	0.9845	0.9647	0.9504	0.9743	0.9738
KA07	0.9997	0.9301	0.9159	0.9579	0.9370
KA08	0.9869	0.8515	0.9378	0.9595	0.9439
KI01	0.9907	0.8882	0.9350	0.9212	0.9670
KI03	0.9359	0.9776	0.9310	0.9630	0.9886
KI04	0.9589	0.9844	0.9573	0.9816	0.9766
KI07	0.9915	0.7866	0.9443	0.8740	0.9387
Average F1 Score	**0.9793**	**0.9065**	**0.9422**	**0.9438**	**0.9387**
Training Time (s)	**165.3350**	**949.2147**	**1333.6493**	**2016.3115**	**1944.5195**

**Fig. 19:**
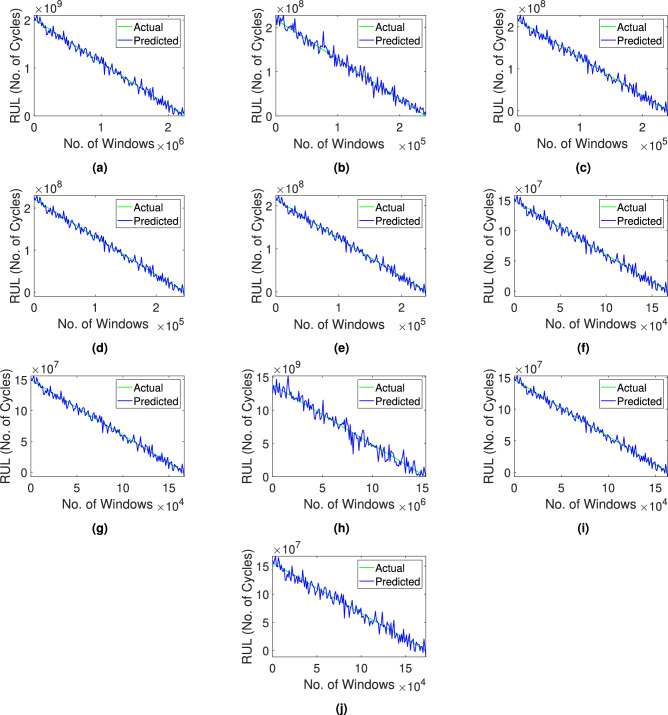
Prediction of the remaining service life of (**a**) KA01, (**b**) KA03, (**c**) KA04, (**d**) KA05, (**e**) KA07, (**f**) KA08, (**g**) KI01, (**h**) KI03, (**i**) KI04 and (**j**) KI07 bearings in PU dataset using the proposed network.

**Fig. 20:**
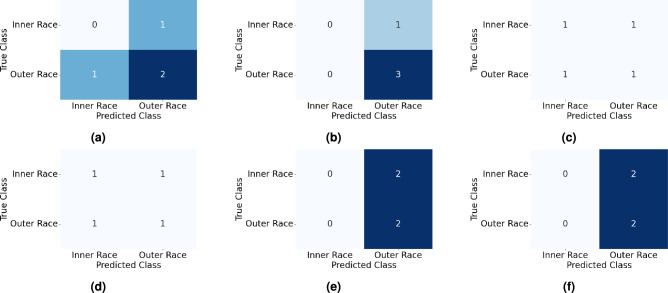
Confusion matrices for prediction of fault types in PU dataset using (**a**) SAM-ANN, (**b**) SAM-CNN, (**c**) SAM-LeNet5, (**d**) SAM-ResNet50, (**e**) SAM-AlexNet, and (**f**) SAM-GoogLeNet.

### Experiment 2: PU dataset

The bearings used in this dataset are a deep groove ball bearing type with inner diameter, outer diameter and width of 17, 40 and 12 mm, respectively^72^. In this study, 10 bearing vibration signals are considered with six outer raceway and four inner raceway failures (https://www.kaggle.com/datasets/alejandromejiao/paderborn). The bearings are operated on a test rig at 900 rpm with a load torque of 0.7 N-m and a radical force of 1 kN. The bearing data, *viz.* KI01, KI03, KI04 and KI07 failed due to inner race fault whereas KA01, KA03, KA04, KA05, KA07 and KA08 suffered damage in the outer raceway. In this case, the dataset has limited and imbalanced failure scenarios but without any mixed failure types. Similarly to the previous experiment, the sensitivity of the features in the time and frequency domains is assessed following the linear correlation as shown in Fig. 18. In this case, $\gamma _6$, $\gamma _7$, LF and $\mu _{\omega }$ are observed as the least significant parameters due to low correlation factors (i.e. $< 0.20$) and subsequently, these features are screened from the input parameters for better efficiency. Overall, the sensitivity analysis of the input time and frequency domain features indicate that the higher order statistical moments (such as hyper-tailedness and above), latitude factor and mean value in frequency domain are insignificant for fault diagnosis. Additionally, an ablation experiment is performed with the PU dataset as presented in Table 13. The results strengthen the choice of window size in the pre-processing and the improvements due to the proposed CNN-GRU network for prognosis. The identification of the change point suffers due to the larger window size *w* but it contradicts accuracy and computational efficiency in Stage-1. In this case, the earlier adopted value of 64 seems to perform well with a better F1 score and limited computational effort. Similarly, the training time required by the networks (i.e., CNN, GRU, transformer and CNN-Transformer) for remaining service life prediction is more than the combined CNN-GRU architecture proposed in Stage-2. The consistency of the results of the datasets considered in this study establishes the advantage of the present proposal.

Following the PAA and SAA, the vibration signals are pre-processed in a similar way as mentioned earlier by considering a window size of 64. The bearing health classification is performed following the Stage-1 process of the proposed framework. Table 14 evaluates the performance of the voting ensemble which provides an average F1 score (approx. 0.979) better than the ANN, LSTM, autoencoder and sparse autoencoder models. Also, the proposed model demonstrates computational efficiency in training (i.e. 165 s) to predict the classification of bearing health. Stage-2 of the proposed network is performed to estimate the bearing service life after identifying the change point and the bearing health. The CNN-GRU model results in average MAE, RMSE and R2 values of 0.035, 0.045 and 0.977, respectively, for RUL prediction of PU dataset bearings. The comparison of the predicted and actual RUL of the bearings is shown in Fig. 19. The quality of prediction remains comparable to the results of the XJTU-SY dataset and better than the state-of-the-art methods discussed earlier. In the third stage, the signals are converted into 2D spectrogram images followed by two iterations of masking based on SAM. A total of six bearing cases are used to train the deep networks (i.e., ANN, CNN, LeNet5, ResNet50, AlexNet and GoogLeNet) and rest are employed to test the performance. Fig. 20 shows the prediction quality of deep networks in identifying the type of bearing fault. Similar results are observed in the case of PU dataset, where SAM-CNN achieves the highest F1 score of approx. 85% among the other deep networks [such as ANN (75%), LeNet5 (50%), ResNet50 (50%), AlexNet (50%) and GoogLeNet (50%)] used in this study. The F1 score has improved to 85% in this dataset compared to the previous experiment. This may be possibly due to the lack of mixed failure cases in this dataset. Also, the accuracy is expected to improve with more data or cases for training. The authors wish to address this issue using data augmentation to improve the fault diagnosis and prognosis in future research. Further, the results strengthen the observations of the previous experiment, where the models such as LeNet5, ResNet50, AlexNet and GoogLeNet predominately predict particular type of failure. In this case, these models overwhelmingly predict the outer race bearing fault.

Both bearing experiments performed using the benchmark datasets illustrate the advantage of using the proposed e-CNN-GRU-SAM framework in generalized applications. It adopts a single framework to improve the bearing fault diagnosis and prognosis (including classification of health, estimation of remaining service life and identification of fault type), which is otherwise not addressed in the literature. The vibration datasets offer limited and imbalanced cases of different failures, with a few cases suffering multiple causes of bearing failure. Further, the training is conducted using these limited samples and the performance is established based on the new untrained data. In addition, the results provide computational efficiency of the proposed framework with the judicious use of the network for specific tasks. Hence, the present experiments strengthen the generalized nature of the proposed network for multiple tasks of fault diagnosis.

## Summary

Condition assessment and prognostic analysis play a vital role in reducing downtime in bearing operations, which indeed helps to improve efficiency. This paper presents a novel three-stage fault diagnosis framework to identify the health, prediction of remaining service life and type of failure in the bearings. The proposed e-CNN-GRU-SAM network combines different learning networks including voting ensemble, CNN, GRU and SAM for forensic analysis and prediction of bearing fault. Bearing vibration signals are pre-processed using PAA and SSA to reduce noise and dimensionality of data without marginalizing useful features. The learning models are trained using different time and frequency parameters along with the detection of change points to establish the bearing health index. The contributions are validated on the basis of two benchmark problems simulating induced and real bearing failures. The training is carried out using limited dataset samples, while the performance is determined using untrained data. Additionally, sensitivity study and ablation experiments are performed to tune the proposed network for better accuracy and efficiency. Based on the results presented, the primary contributions of the proposed work are as follows:

- A novel multi-stage deep learning network is proposed for efficient condition assessment and prognosis in a unified framework. This hybrid network is developed combining advantages of different prediction models (i.e. voting ensemble, CNN, GRU and SAM) for generalized applications.
- The voting ensemble based classification of the bearing health results in nearly accurate detection of the change point following second-order moment. Similarly, a hybrid architecture combining the GRU network with CNN demonstrates improvement in bearing RUL prediction. The proposed network performs better than the recently developed deep networks such as DANN, TCN-SA, ResNet-MA, GRU-DeepAR, CAMTCN, SACGNet, CARDenseNet and TCN-transformer for bearing prognostics.
- The present study shows an improvement in the identification of bearing failure types using the novel SAM with deep network learning. The time series is converted into a 2D visual spectrogram modified with zero-shot spatial-temporal masks using multiple iterations to classify the distinct features in the segmented image data. This improves training of different deep networks (i.e., ANN, CNN, LeNet5, ResNet50, AlexNet and GoogLeNet) where CNN architecture achieves better diagnostic performance in predicting the type of bearing failure.

The present study deals with consistent condition experiments and hence, the affect of imbalance in data, uncertainty and/or variation in operating conditions needs further assessment, which the authors wish to address in future research. Overall, these contributions highlight the strong potential of stage-wise and time-frequency masked visual data training in the forensic analysis of faults and continuous bearing monitoring.

## Data Availability

Data supporting the findings of this study are available from A.K.R. upon reasonable request.
